# SpxA1 and SpxA2 Act Coordinately To Fine-Tune Stress Responses and Virulence in *Streptococcus pyogenes*

**DOI:** 10.1128/mBio.00288-17

**Published:** 2017-03-28

**Authors:** Gary C. Port, Zachary T. Cusumano, Paul R. Tumminello, Michael G. Caparon

**Affiliations:** Department of Molecular Microbiology, Washington University School of Medicine, Saint Louis, Missouri, USA; University of Mississippi Medical Center

## Abstract

SpxA is a unique transcriptional regulator highly conserved among members of the phylum *Firmicutes* that binds RNA polymerase and can act as an antiactivator. Why some *Firmicutes* members have two highly similar SpxA paralogs is not understood. Here, we show that the SpxA paralogs of the pathogen *Streptococcus pyogenes*, SpxA1 and SpxA2, act coordinately to regulate virulence by fine-tuning toxin expression and stress resistance. Construction and analysis of mutants revealed that SpxA1^−^ mutants were defective for growth under aerobic conditions, while SpxA2^−^ mutants had severely attenuated responses to multiple stresses, including thermal and oxidative stresses. SpxA1^−^ mutants had enhanced resistance to the cationic antimicrobial molecule polymyxin B, while SpxA2^−^ mutants were more sensitive. In a murine model of soft tissue infection, a SpxA1^−^ mutant was highly attenuated. In contrast, the highly stress-sensitive SpxA2^−^ mutant was hypervirulent, exhibiting more extensive tissue damage and a greater bacterial burden than the wild-type strain. SpxA1^−^ attenuation was associated with reduced expression of several toxins, including the SpeB cysteine protease. In contrast, SpxA2^−^ hypervirulence correlated with toxin overexpression and could be suppressed to wild-type levels by deletion of *speB*. These data show that SpxA1 and SpxA2 have opposing roles in virulence and stress resistance, suggesting that they act coordinately to fine-tune toxin expression in response to stress. SpxA2^−^ hypervirulence also shows that stress resistance is not always essential for *S. pyogenes* pathogenesis in soft tissue.

## IMPORTANCE

For many pathogens, it is generally assumed that stress resistance is essential for pathogenesis. For *Streptococcus pyogenes*, environmental stress is also used as a signal to alter toxin expression. The amount of stress likely informs the bacterium of the strength of the host’s defense response, allowing it to adjust its toxin expression to produce the ideal amount of tissue damage, balancing between too little damage, which will result in its elimination, and too much damage, which will debilitate the host. Here we identify components of a genetic circuit involved in stress resistance and toxin expression that has a fine-tuning function in tissue damage. The circuit consists of two versions of the protein SpxA that regulate transcription and are highly similar but have opposing effects on the severity of soft tissue damage. These results will help us understand how virulence is fine-tuned in other pathogens that have two SpxA proteins.

## INTRODUCTION

Low-pH environments, oxidative stress, nutritional starvation, and fluctuations in temperature are a few of the many stresses encountered by both pathogenic and environmental bacteria. To protect themselves against these stresses, bacteria employ numerous countermeasures via both general and stress-specific mechanisms. Examples of the former include alteration of membrane fluidity and degradation of damaged or misfolded proteins. For the latter, examples include scavengers of reactive oxygen species, differential regulation of heat shock chaperones, and alternative catabolic pathways (reviewed in reference 1). Since the various stresses imposed by the host’s defensive responses can act as markers of specific temporal stages of disease progression or host compartments, pathogens often coordinately regulate stress response programs with virulence factor expression (2). Understanding these coregulatory networks can provide insight into the specific cues used to regulate of virulence factor expression in temporal and/or tissue-specific patterns.

Because of the diversity of host tissues that it can infect and the types of diseases that it can cause, host defensive responses challenge *Streptococcus pyogenes* with numerous environmental stresses. This Gram-positive bacterium primarily infects soft tissues of the pharynx and skin to cause diseases that include pharyngitis (strep throat) and impetigo to more severe diseases such as necrotizing fasciitis and streptococcal toxic shock syndrome (3). However, the bacterium can also infect the eyes, ears, lungs, muscles, bloodstream, lymph nodes, subcutaneous tissues, and perianal and vaginal tissues (4). The later stages of infection in the majority of these tissues are characterized by intense inflammation accompanied by high fever, which can impose considerable oxidative, thermal, and cell envelope stresses. There is considerable evidence that *S. pyogenes* senses several of these stresses to regulate virulence factor expression. For example, sublethal concentrations of host cationic antimicrobial peptides (CAPs) can induce changes in the expression of multiple virulence genes by several mechanisms, including recognition by a two-component transcription regulator (5) and disruption of the ExPortal, a discrete membrane microdomain required for optimal protein secretion and processing (6).

To gain insight into how stress sensing by the ExPortal is coupled to virulence factor expression, we recently conducted a genetic screening to identify mutations that confer resistance to the disruptive effects of CAPs (7). Loss of function due to mutations in the gene encoding ClpX exhibited multiple phenotypes, including increased resistance to killing by the CAP polymyxin B, enhanced resistance to ExPortal disruption, resistance to heat stress, and decreased expression of SpeB (7), a secreted cysteine protease important for virulence (reviewed in reference 8). ClpX is a member of a family of AAA+ ATPases that interact with ends of the multimeric barrel-shaped serine protease ClpP and are responsible for the recognition, unfolding, and translocation of proteins into the proteolytic core (reviewed in references 9 and 10). All streptococci encode ClpX, ClpL, ClpE, and ClpC, each of which recognizes a distinct sequence motif on proteins destined for degradation (11). The ClpP proteolytic subunit is essential for growth under stress conditions in a variety of Gram-positive bacteria (12–14), including various streptococcal species (11, 15, 16).

Clp ATPases typically contribute to stress resistance by removing or refolding damaged proteins. However, ClpX has a nonstress role in the regulation of many complex processes in streptococci and other Gram-positive bacteria with low GC content. In these species, mutation of ClpX and/or ClpP typically results in pleiotropic effects, including defects in competence, sporulation, cell division, and the regulation of certain stress-specific responses (17). These defects are often alleviated through secondary mutation of the ClpXP substrate SpxA (suppressor of *clpP* and *clpX*) (reviewed in references 18 and 19). SpxA has been most thoroughly characterized in *B. subtilis*, where it has been shown to act as a global transcriptional regulator through its interaction with the α subunit of RNA polymerase (RNAP) (20). SpxA has been proposed to function primarily as an antiactivation factor by interfering with the ability of transcriptional activators to bind to RNAP (20). However, there is evidence that it may also directly influence the interaction of RNAP with DNA, resulting in both positive and negative regulation of >100 genes in *B. subtilis* (21, 22). Since SpxA is a ClpX substrate, the absence of ClpXP chaperone or proteolytic activity results in a dramatic increase in the level of SpxA. Thus, the loss of SpxA can often suppress phenotypes associated with the loss of ClpXP (18, 19). While several Gram-positive bacterial species with low GC content encode a single version of SpxA, all members of the family *Streptococcaceae* possess two paralogs typically named SpxA1 and SpxA2 (23–25).

In this study, we examined various SpxA1 and SpxA2 mutants to further probe the mechanism by which ClpX simultaneously regulates both stress response and virulence factor expression in *S. pyogenes*. This analysis revealed nonoverlapping roles for each of the SpxA proteins in stress resistance, toxin expression, and pathogenesis, suggesting that they act coordinately to fine-tune these responses. Unexpectedly, SpxA2^−^ mutants were hypervirulent despite being profoundly sensitive to thermal and oxidative stresses, revealing that sensitivity to these stresses does not hinder the ability of *S. pyogenes* to infect soft tissue.

## RESULTS

### SpeB regulation requires both ClpX and ClpP.

**FIG 1:**
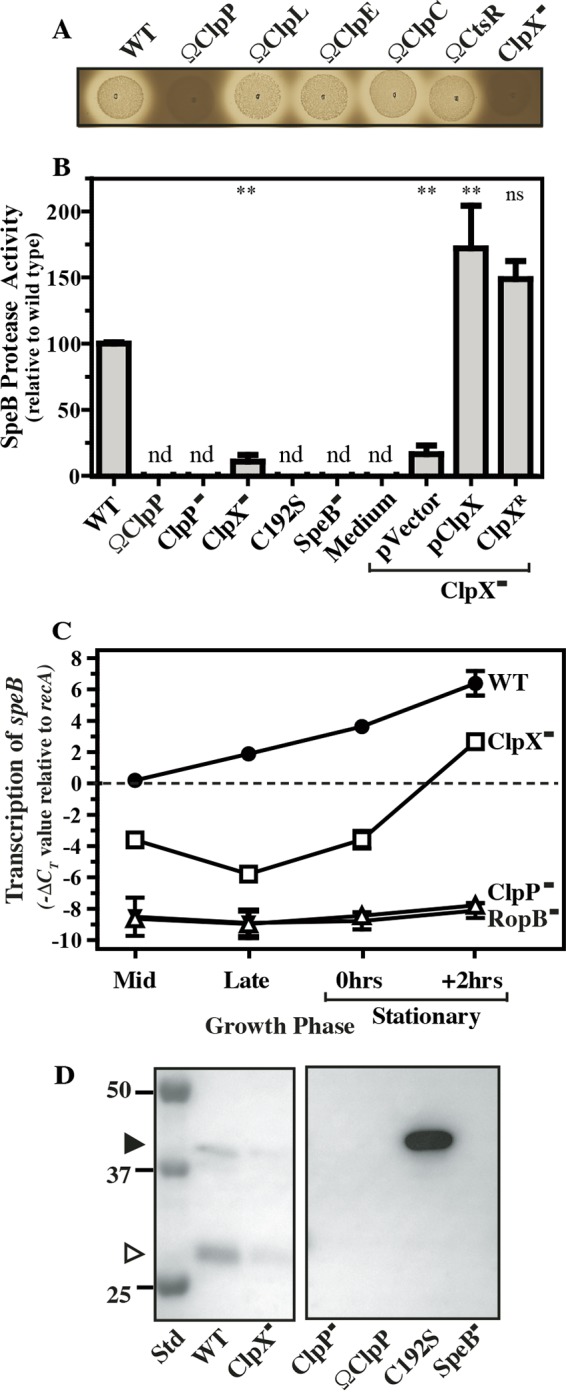
ClpXP positively regulates SpeB expression and activation. The *S. pyogenes* strains indicated were tested for protease activity by spotting onto protease indicator plates (A) and by determination of protease activity in cell-free supernatants with a fluorescent casein substrate (B). For indicator plates, images were captured after 16 h of incubation. The diameter of the zone of clearing around bacterial growth is an indication of the level of protease activity. For supernatants, nd indicates that the level of activity was below the limit of detection (<1.0% of that of the WT). For selected strains, the relative levels of the *speB* transcript over the course of growth was determined by real-time RT-PCR (C) and the efficiency of processing of the proSpeB zymogen (40 kDa, closed arrowhead) into catalytically active SpeB protease (27 kDa, open arrowhead) was determined by Western blot analysis of culture supernatants at 2 h after entry into stationary phase (D). The migration of several molecular weight standards (Std, in kilodaltons) is shown on the left. The C192S mutant strain expresses catalytically inactive SpeB, and the SpeB^−^ mutant strain has a deletion of *speB*. The data shown are representative of at least two independent experiments and, where indicated, represent the mean and standard error of the mean derived from duplicate determinations of samples from at least two independent experiments (**, *P* < 0.01; ns, not significant).

ClpX^−^ mutants fail to express SpeB protease activity and display significantly decreased levels of the *speB* transcript (7). To determine if additional Clp proteins participate in SpeB regulation and whether ClpX acts in a ClpP-dependent or -independent manner, mutants were generated by a gene insertion strategy in *clpP*, the genes for the three additional members of the Clp chaperone family (ClpC, ClpE, and ClpL), and a regulator of the transcription of these genes (CtsR) that are found in serotype M14 *S. pyogenes* HSC5 (see Table S1). CtsR is predicted to bind to a conserved DNA sequence within the promoters of *clpP*, *groES-groEL*, and the genes encoding all Clp chaperones, with the exception of ClpX (see Fig. S1 in the supplemental material). Of the Clp chaperones, only the loss of ClpX was associated with a complete loss of proteolytic activity when examined on protease indicator plates and a 90% reduction relative to the wild type (WT) as measured by a protease assay (Fig. 1A and B). This loss could be complemented by the expression of ClpX from a plasmid or from an ectopic chromosomal locus (Fig. 1B). Insertional inactivation of *clpP*, as well as a nonpolar mutation in *clpP* (ΔClpP::*aad9*), resulted in a failure to detect any protease activity, similar to a SpeB^−^ mutant, a catalytically inactive C192S allele of *speB*, and medium alone (Fig. 1A and B). Investigation of transcript levels indicated that while ClpX and ClpP both affect SpeB expression over time, they do so in different patterns. SpeB expression is subject to multiple levels of regulation (reviewed in reference 8), shows a strict dependence on the growth phase, and is induced only upon entry into stationary phase (26), and its transcription is dependent on the DNA-binding transcriptional activator RopB (27). Analysis of *speB* transcript levels by real-time reverse transcription (RT)-PCR indicated that *speB* was expressed in the ClpX^−^ mutant, yet its induction was delayed and transcript levels were decreased at all of the time points investigated compared to those of the WT, including 2 h after entry into stationary phase, when *speB* is maximally expressed in the WT (Fig. 1C). In contrast, the loss of ClpP phenocopied the loss of RopB, in that *speB* expression was undetectable throughout the time course (Fig. 1C). Similar differences were observed upon examination of the SpeB polypeptide in culture supernatants. Following its secretion, SpeB is proteolytically processed via multiple cleavage events from a 40-kDa zymogen into a 28-kDa active protease. A Western blot analysis of culture supernatants indicated that while the ClpX^−^ mutant produced less SpeB than the WT (Fig. 1D), SpeB was undetectable in the absence of ClpP, similar to a SpeB^−^ mutant, in contrast to the catalytically inactive C192S SpeB mutant protein, which remains in its unprocessed 40-kDa zymogen form (Fig. 1D).

10.1128/mBio.00288-17.1 FIG S1  Predicted CtsR binding sites in *S. pyogenes*. (A) CtsR consensus binding sequence derived from 68 predicted CtsR binding sites from a variety of streptococcal and lactococcal species. (B) CtsR consensus binding sequence derived from five predicted *S. pyogenes* CtsR binding sites. (C) The 5′ promoter regions of the genes indicated are displayed as sense strands, with the exception of *ctsR*, which is displayed as an antisense strand. The promoter regions of the genes indicated are marked to include putative −35 and −10 transcriptional sites (underlined), CtsR binding sites (red), invariant residues (uppercase), and ATG start codons (italicized) are indicated. Polycistronic genes are indicated with hyphens. Download FIG S1, PDF file, 0.2 MB. Copyright © 2017 Port et al. 2017 Port et al. This content is distributed under the terms of the Creative Commons Attribution 4.0 International license.

10.1128/mBio.00288-17.6 TABLE S1  Bacterial strains used in this study. Download TABLE S1, PDF file, 0.1 MB. Copyright © 2017 Port et al. 2017 Port et al. This content is distributed under the terms of the Creative Commons Attribution 4.0 International license.

### **SpxA2 negatively regulates SpeB**.

**FIG 2:**
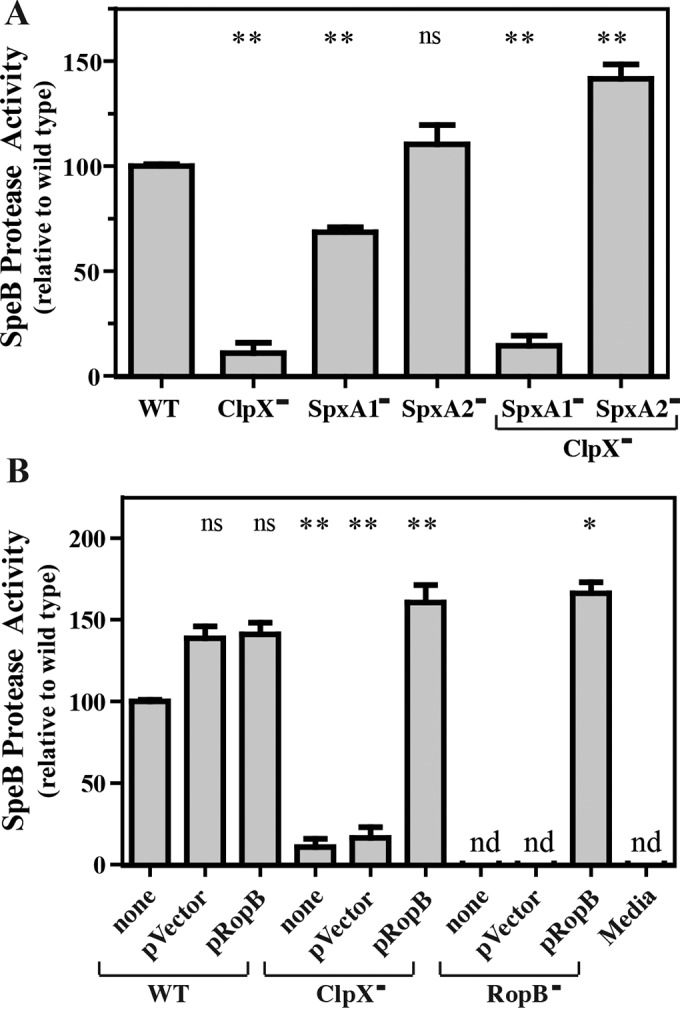
SpxA2 negatively regulates SpeB via RopB. Protease activity in cell-free culture supernatants was determined as described for Fig. 1 in various ClpX^−^ and SpxA^−^ mutants (A) and in the strains indicated overexpressing RopB (B). For the latter, RopB was overexpressed from a plasmid (pRopB) for comparison to the strain with no plasmid (none) or the vector alone (pVector). The abbreviation nd indicates that activity was below the limit of detection (<1.0% relative to the WT). The data shown are the means and the standard errors of the means derived from duplicate determinations of samples from at least two independent experiments (*, *P* < 0.05; **, *P* < 0.01; ns, not significant).

Like all streptococci, *S. pyogenes* contains two paralogs of the gene for SpxA, whose functional loss can suppress many of the complex phenotypes that result from a ClpX deficiency (reviewed in references 18 and 19). Both Spx paralogs possess a conserved N-terminal CxxC redox-sensing domain and a conserved glycine residue (see Fig. S2) critical for its interaction with the C-terminal domain of the α subunit (αCTD) of RNAP (20). Multiple comparisons show that *S. pyogenes* SpxA1 is most similar to Spx in those bacterial species that have only a single version of Spx (see Fig. S2). Mutants were constructed with in-frame deletions in each Spx-encoding gene, in both the WT and ClpX^−^ backgrounds (see Table S1). Double mutants were then tested for the ability of Spx mutations to suppress the SpeB expression defect of a ClpX^−^ mutant. Analysis of proteolytic activity revealed that the loss of SpxA1 did not alter the ClpX^−^-associated SpeB expression defect. In contrast, the ClpX^−^ SpxA2^−^ mutant not only suppressed the defective ClpX^−^ phenotype but expressed SpeB at levels significantly higher than those of the WT strain (Fig. 2A). Furthermore, in the single mutant strains, SpeB expression was significantly reduced in the SpxA1^−^ mutant but was similar to that in the WT in the SpxA2^−^ mutant (Fig. 2A). Together, these data indicate that SpxA2 acts as a negative regulator of SpeB, whose repressive activity is antagonized by ClpX. Furthermore, since SpxA1 and SpxA2 have the opposite effect on SpeB expression and may compete for binding to αCTD, these data indicate that they may act antagonistically. These effects were not limited to SpeB, as it was found that the ClpX^−^ mutant also had reduced transcription of the gene encoding a second secreted toxin, the cytolysin streptolysin O, that could be suppressed by the loss of SpxA2 but not by the loss of SpxA1 (see Fig. S3A).

10.1128/mBio.00288-17.2 FIG S2  Alignment of SpxA1 and SpxA2 amino acid sequences from *S. pyogenes* and other Gram-positive bacteria. Alignment of sequences of SpxA1 and SpxA2 from *S. pyogenes* (spy and spyh), *S. mutans* (smu) (1), *S. pneumoniae* (spn) (2), *S. sanguinis* (ssa) (3), *Lactococcus lactis* (lla) (4), *Enterococcus faecalis* (efa) (5), *Staphylococcus aureus* (sau) (6), *Bacillus subtilis* (bsu) (7), and *Listeria monocytogenes* (lmo) (8) was produced by ClustalO. Symbols indicating that residues are identical (*), conservative (:), semiconservative (.), or nonconservative (no symbol) are located above the residues for comparison of SpxA1^−^ or SpxA2^−^homologues and below the residues for the comparison of all sequences. Boxed residues include the N-terminal CxxC redox-sensing domain and the G52 residue critical for interaction with RNAP in *B. subtilis*. Download FIG S2, PDF file, 0.1 MB. Copyright © 2017 Port et al. 2017 Port et al. This content is distributed under the terms of the Creative Commons Attribution 4.0 International license.

10.1128/mBio.00288-17.3 FIG S3  ClpX positively regulates *slo* expression, and overexpression of SpxA2, but not SpxA1, suppresses the expression of SpeB. (A) The expression of the gene encoding streptolysin O (L897_00955) in the mutants indicted was analyzed by real-time RT-PCR as previously described (9) at the mid-logarithmic (50% of final OD_600_) or late-logarithmic (75% of final OD_600_) phase of growth in C medium. The data presented represent the mean and standard error of the mean derived from at least three independent experiments. (B) The plasmid vector alone (pVector) or the vector expressing SpxA1 (pSpxA1) or SpxA2 (pSpxA2) (see Text S1) was introduced into the WT strain (HSC5). Overnight cultures were serially diluted, and aliquots were plated on protease indicator medium (dilution) and imaged following an additional 24 h of incubation, as shown. SpeB protease activity is apparent as a zone of clearing around the area of bacterial growth. As previously described (5), the relative level of SpeB expression corresponds to the highest dilution at which a zone of clearing is observed. Strains: pVector, GCP017; pSpxA1, ZC572; pSpxA2, ZC573 (see Table S1). Download FIG S3, PDF file, 0.7 MB. Copyright © 2017 Port et al. 2017 Port et al. This content is distributed under the terms of the Creative Commons Attribution 4.0 International license.

10.1128/mBio.00288-17.10 TEXT S1  Supplemental materials and methods and references. Download TEXT S1, DOCX file, 0.03 MB. Copyright © 2017 Port et al. 2017 Port et al. This content is distributed under the terms of the Creative Commons Attribution 4.0 International license.

### SpxA2 antagonizes RopB.

Numerous regulators have been reported to affect the expression of SpeB (8). However, transcription of *speB* has an absolute dependence on the transcription activator RopB, a member of the Rgg family of DNA-binding proteins (27). Since SpxA canonically functions as an antiactivator, we tested the hypothesis that SpxA2 antagonizes the essential activity of RopB for activation of *speB* transcription. This hypothesis predicts that SpxA2 and RopB are in competition for binding to RNAP and that SpxA2 inhibits the ability of RopB to recruit RNAP to the *speB* promoter. This model predicts that the level of SpeB expression will be sensitive to the ratio of the two regulators. To test this, *ropB* was overexpressed from an ectopic promoter on a multicopy plasmid, which complemented a RopB^−^ mutant to produce significantly more SpeB than the WT, while the activity of the RopB^−^ mutant alone or with the empty vector was similar to that of uninoculated medium (Fig. 2B). Consistent with this hypothesis, overexpression of *ropB* in the ClpX^−^ mutant suppressed its SpeB expression defect, resulting in overexpression of SpeB similar to that of the complemented RopB^−^ mutant (Fig. 2B). Also consistent was the finding that ectopic overexpression of SpxA2 in the WT resulted in repression of SpeB expression (see Fig. S3B).

### **ClpXP regulates multiple stress resistances via SpxA2**.

**FIG 3:**
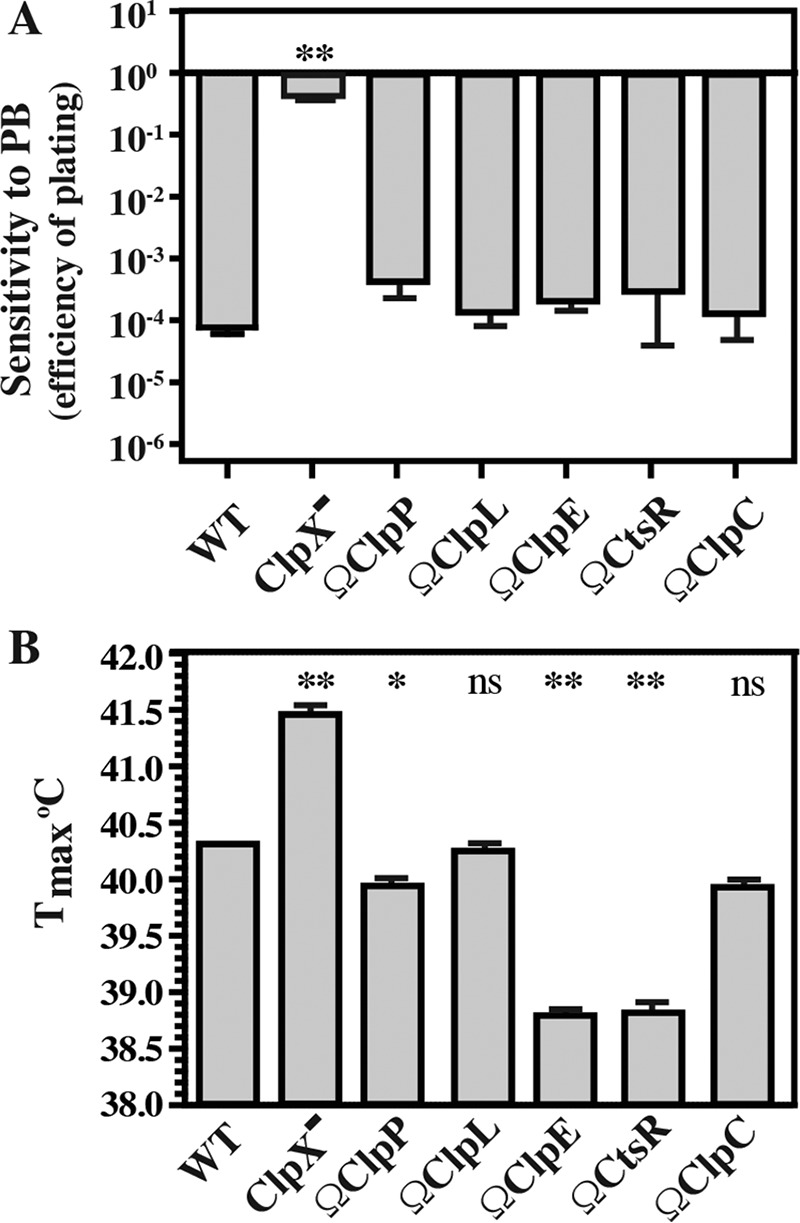
ClpX^−^, but not other Clp mutants, displays PB^r^ and heat resistance. The strains indicated were monitored for PB^r^ (A), and the *T*_max_ was determined (B). PB^r^ is reported as the efficiency of plating on medium lacking or containing polymyxin B (40 μg ml^−1^) and was calculated as log_10_ (CFU_+PB_/CFU_unmod_). The *T*_max_ was determined by incubating strains over a 0.1°C stepwise gradient and scoring them for growth after 24 h. The data shown represent means and standard errors of the means derived from duplicate determinations of samples from at least three independent experiments (*, *P* < 0.05; **, *P* < 0.01; ns, not significant).

**FIG 4:**
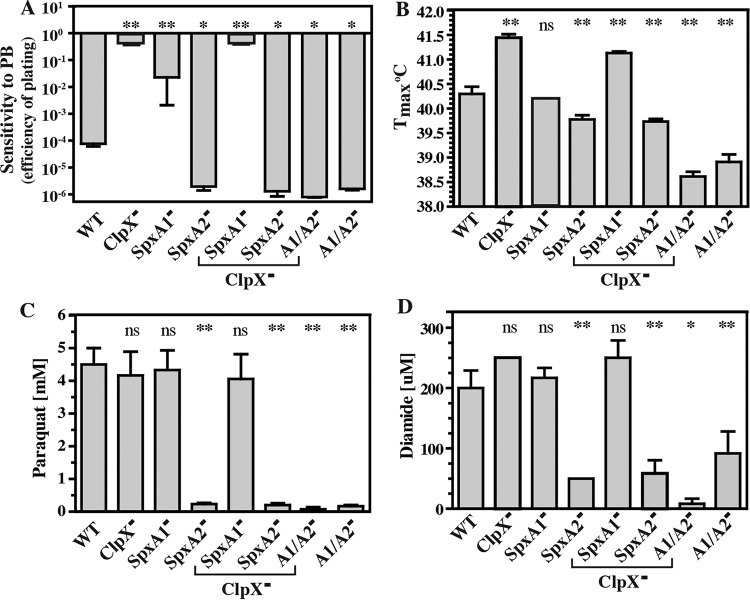
ClpX negatively regulates multiple stress resistances via SpxA2. The strains indicated were monitored for PB^r^ (A), for the *T*_max_ (B), and for resistance to the oxidizing agents paraquat (C) and diamide (D). PB^r^ was determined as efficiency of plating on medium lacking or containing polymyxin B (40 μg ml^−1^) and calculated as log_10_ (CFU_+PB_/CFU_unmod_). The *T*_max_ was measured by incubating strains over a 0.1°C stepwise gradient and scoring them for growth after 24 h. Resistance to paraquat and diamide was calculated as the lowest concentration of reagent that resulted in a <10% final growth yield of the treated versus untreated strain. The data shown are the means and standard errors of the means derived from duplicate determinations from at least three independent experiments (*, *P* < 0.05; **, *P* < 0.01; ns, not significant).

SpxA has been shown in other organisms to regulate stress responses via ClpXP (17), and we have reported that the ClpX^−^ mutant displays greater resistance to polymyxin B (PB^r^) and heat stress than the WT (7) (also see Fig. S4). To expand this analysis, the various Clp, Spx, and regulatory mutants were tested for the ability to grow under a variety of stress conditions. Only the ClpX^−^ mutant displayed increased PB^r^ (Fig. 3A) and heat resistance (Fig. 3B), and unlike SpeB regulation (Fig. 1A), neither of these phenotypes required ClpP (Fig. 3). For PB^r^, SpxA1 and SpxA2 had an antagonistic relationship. The SpxA1^−^ mutant was more resistant than the WT, while the SpxA2^−^ mutant was more sensitive (Fig. 4A). The effect of the SpxA2^−^ mutation on stress sensitivity was dominant when tested in combination with ClpX and SpxA1 mutations (Fig. 4A), and SpxA2 also had a predominant role in thermal tolerance, as SpxA2^−^ mutants had a lower maximum growth temperature both individually and when tested in combination with SpxA1 mutations (Fig. 4B). Although the loss of SpxA1 had no effect, the loss of both SpxA1 and SpxA2 was combinatorial, resulting in even greater thermal sensitivity (Fig. 4B). Considering that SpxA mutants in other bacterial species display differential resistance to oxidative stress (22, 28, 29), the *S. pyogenes* mutants were challenged by the addition of oxidizing agents to the medium. While the ClpX^−^ mutant did not display increased resistance, all mutants lacking SpxA2 had significantly lower MICs of both paraquat (Fig. 4C) and diamide (Fig. 4D). Together, these data indicate that stress resistance is mediated primarily via SpxA2 and suggest that the increase in stress resistance displayed by the loss of ClpX results from increased levels of SpxA2.

10.1128/mBio.00288-17.4 FIG S4  Growth under aerobic conditions is bacteriostatic. The strains indicated were cultured in liquid medium for 48 h under anaerobic (minus O_2_) or aerobic (plus O_2_) conditions, and growth was measured by determination of CFU counts. After the first 24 h of growth, conditions were either shifted or left the same, as indicated at the right (A, anaerobic to anaerobic; B, anaerobic to aerobic; C, aerobic to aerobic; D, aerobic to anaerobic). Download FIG S4, PDF file, 0.1 MB. Copyright © 2017 Port et al. 2017 Port et al. This content is distributed under the terms of the Creative Commons Attribution 4.0 International license.

### **SpxA1 and SpxA2 are required for aerobic growth**.

**FIG 5:**
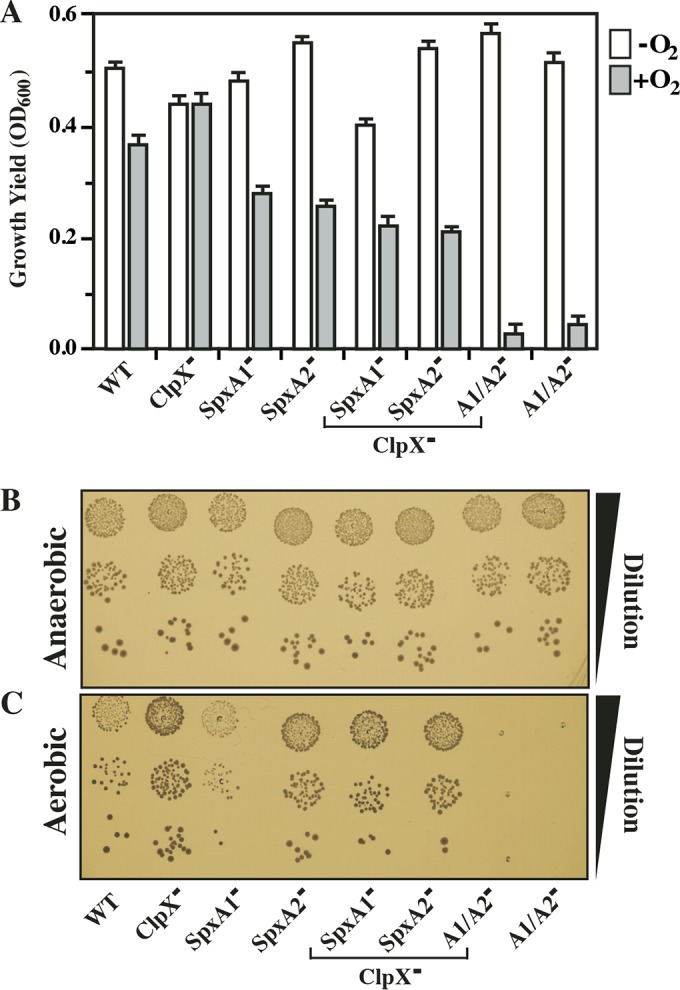
SpxA1 is required for aerobic growth. The strains indicated were tested for growth under anaerobic (minus O_2_) or aerobic (plus O_2_) conditions in liquid medium (A) and for the ability to form colonies on solid medium (B and C). For liquid medium, growth was measured by determining the OD_600_ following 24 h of incubation. For solid medium, overnight liquid cultures were serially diluted (Dilution) and then plated under aerobic or anaerobic conditions for examination after 24 h. The data shown are the means and standard errors of the means derived from duplicate determinations of at least three independent experiments or are images representative of at least three independent experiments.

Under anaerobic conditions, all mutants had growth yields comparable to those of the WT in liquid medium (Fig. 5A) and formed colonies on solid medium (Fig. 5B) equivalent to those of the WT. However, under aerobic conditions, the loss of either SpxA1 or SpxA2 alone resulted in a 40 to 60% decrease in the final growth yields and a combination of the two mutations resulted in a >90% reduction of the yield in liquid medium (Fig. 5A) and an inability to form colonies at all on solid medium (Fig. 5C). In contrast, the ClpX^−^ mutant exhibited robust growth under all conditions and suppressed the SpxA1^−^ growth defect on solid medium (Fig. 5A to C). A series of cultures were then shifted between conditions. When liquid cultures of the various SpxA mutants were grown under anaerobic conditions for 24 h and subsequently exposed to aerobic conditions, no decrease in the CFU count was observed (see Fig. S4A and B). However, when cultures grown under aerobic conditions were then exposed to anaerobic conditions, the CFU count increased over the course of an additional 24 h to obtain final densities similar to those of the WT (see Fig. S4C and D). These data suggest that the inhibition of aerobic growth mediated by loss of SpxA1 or SpxA2 is likely to occur via a bacteriostatic rather than bactericidal mechanism.

### Loss of SpxA2 results in hypervirulence.

**FIG 6:**
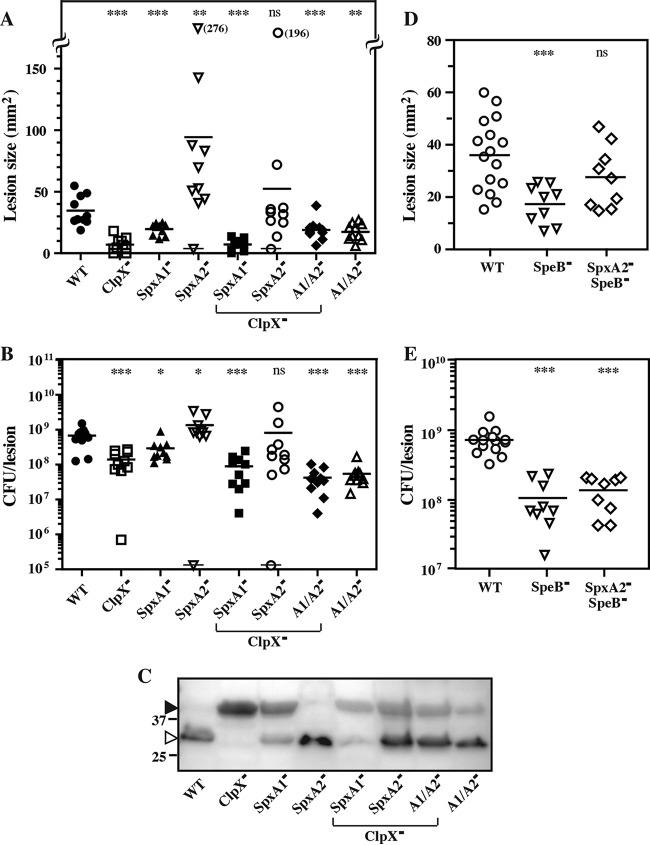
SpxA1^−^ mutants are attenuated, and SpxA2^−^ mutants are hypervirulent. Hairless SKH1 mice were infected subcutaneously with 10^7^ CFU of the strains indicated. The areas of the resulting ulcers at day 3 postinfection are shown (A, D). Each symbol represents an individual animal. A symbol overlaid with a horizontal line indicates a mouse that succumbed to infection prior to day 3. Values in parentheses adjacent to symbols placed off scale indicate actual lesion areas. Tissues were then harvested and processed to determine CFU counts (B, E) and levels of SpeB (C), which were determined by immunoblotting of samples normalized for bacterial burden. On the left, the open and closed arrowheads indicate processed and unprocessed SpeB, respectively, and the migration of several molecular weight standards (in kilodaltons) is shown. The data are pooled from at least two independent experiments, and the mean is indicated by a bar (*, *P* < 0.05; **, *P* < 0.01; ***, *P* < 0.001; ns, not significant) or are an image representative of three independent experiments.

Since ClpX modulates virulence gene expression via SpxA1 and SpxA2, we assessed the pathogenic potential of these mutants during infection. In the murine subcutaneous infection model, *S. pyogenes* HSC5 (WT) forms a localized lesion characterized by ulceration and the formation of an eschar that expands in size to reach a peak area by day 3 postinfection (4). Loss of ClpX or SpxA1 resulted in a significant decrease in virulence, as measured by both smaller lesion sizes (Fig. 6A) and tissue CFU burdens (Fig. 6B). In contrast, loss of SpxA2 resulted in hypervirulence, with significantly larger lesions and bacterial burdens (Fig. 6A and B), and rapidly developing lesions that were significantly larger as early as 24 h postinfection (see Fig. S5). Loss of SpxA2, but not that of SpxA1, could suppress the virulence defect of the ClpX^−^ mutant and restore virulence to WT levels (Fig. 6A and B). However, mutants lacking both SpxA1 and SpxA2 were attenuated (Fig. 6A and B). Examination of SpeB expression and maturation by a Western blot analysis of tissue homogenates normalized for tissue burden revealed that production of the active form of SpeB *in vivo* paralleled that observed in C medium. Relative to the WT, the ClpX^−^ and SpxA1^−^ mutants produced primarily unprocessed and inactive SpeB, the SpxA2^−^ mutant produced high levels of fully processed SpeB, and the loss of SpxA2, but not that of SpxA1, suppressed the ClpX^−^ defect (Fig. 6C). While there was not a strict correlation between SpeB expression and virulence across the mutant panel, high SpeB expression was a prominent phenotype associated with the loss of SpxA2. Therefore, we tested whether SpeB contributed to SpxA2^−^ hypervirulence. Deletion of *speB* alone resulted in attenuation relative to the WT (Fig. 6D and E). Subsequent deletion of *speB* in a SpxA2^−^ background attenuated hypervirulence to levels at or just below those of the WT (Fig. 6D and E). Taken together, these data show that SpxA1 and SpxA2 have opposing effects on virulence, that SpeB contributes to SpxA2^−^ hypervirulence, and that the loss of SpxA1 is dominant over the loss of SpxA2 since all mutants that lack both genes are attenuated despite elevated SpeB expression.

10.1128/mBio.00288-17.5 FIG S5  SpxA2^−^ hypervirulence is evident early in infection. Hairless SKH1 mice were infected subcutaneously with 10^7^ CFU of the strains indicated. The areas of the resulting ulcers were analyzed 24 h postinfection. The data are pooled from at least two independent experiments, and the mean is indicated by a bar. Differences between WT and mutant strains were tested for significance with the Mann-Whitney test (***, *P* < 0.001; ns, not significant). Download FIG S5, PDF file, 0.1 MB. Copyright © 2017 Port et al. 2017 Port et al. This content is distributed under the terms of the Creative Commons Attribution 4.0 International license.

## DISCUSSION

We previously identified ClpX as a regulator of PB^r^ and ExPortal integrity in serotype M14 *S. pyogenes* HSC5 (7). In the present study, we showed that ClpX mediates PB^r^ via SpxA2, which also regulates multiple stress resistances and toxin expression. SpxA1 and SpxA2 have opposing effects on virulence, with SpxA1 acting as a positive regulator and SpxA2 acting as a negative regulator whose absence results in hypervirulence, suggesting that these two proteins act coordinately to fine-tune virulence in response to various stresses. Remarkably, the SpxA2^−^ mutant was hypervirulent despite its exquisite sensitivity to stress, suggesting that stress resistance is not always essential for the ability of *S. pyogenes* to infect and damage soft tissue.

In many other Gram-positive bacterial species, the ClpXP proteasome regulates the stability of multiple proteins, including the paralogous SpxA family members that function as antiactivators to occlude the interaction of various transcriptional regulators with RNAP. Numerous studies have shown that mutation of SpxA can suppress many phenotypes resulting from the loss of ClpX that arise from reduced turnover of SpxA’s regulatory activities (reviewed in references 18 and 19). What is not understood is why *S. pyogenes* and other streptococcal species have two SpxA family members. Analysis across multiple streptococcal species has revealed that the loss of either SpxA1 or SpxA2 is tolerated, as is the simultaneous loss of both paralogs (23, 29), although exceptions have been noted (24). While both are typically involved in stress responses, the SpxA1 and SpxA2 regulons may be nonoverlapping. For example, the loss of SpxA1 is often (23, 28–31), but not always (24), associated with increased sensitivity to oxidative stress, while the loss of SpxA2 can be pleiotropic, resulting in growth defects and sensitivities to multiple stresses, with phenotypes ranging from mild (29) to severe (23, 24). Taken together, these observations indicate that SpxA1 and SpxA2 are nonredundant and likely regulate different sets of genes.

The analysis of the *S. pyogenes* SpxA^−^ mutants presented here supports this model. With regard to stress, the SpxA^−^ mutants had distinct phenotypes; the SpxA1^−^ mutant lost the ability to grow aerobically, while the SpxA2^−^ mutant was sensitive to oxidative stress. These two phenotypes are not necessarily identical and can involve different types of genes. Resisting oxidative stress requires gene products that can counteract the damage caused by oxidation or can directly detoxify the toxic products resulting from the reduction of molecular oxygen that can be generated by various metabolic or host defense pathways. These include various peroxidases, superoxide dismutases, metal transporters, and DNA repair enzymes (32). The ability to grow under aerobic versus anaerobic conditions also involves enzymes that require molecular oxygen as a cofactor or whose activity is modulated by oxygen. Examples for *S. pyogenes* include NADH oxidase, lactate oxidase, and pyruvate-formate lyase (33). With regard to virulence, the SpxA mutants also had distinct phenotypes, as SpxA1^−^ mutants were attenuated while SpxA2^−^ mutants were hypervirulent. Associated with hypervirulence was overexpression of the SpeB cysteine protease, which can enhance virulence by a number of mechanisms (34). The observation that SpxA2 was also a negative regulator of the secreted SLO cytolysin suggests that SpxA2 may have a more extensive role in the regulation of secreted toxins whose dysregulation may contribute to the hypervirulence phenotype of the SpxA2^−^ mutant. Finally, analysis of SpxA1^−^ SpxA2^−^ double mutants also supports a role for the regulation of distinct gene subsets, as this mutant had properties different from those of either single mutant. For example, SpxA1^−^ attenuation was dominant over SpxA2^−^ hypervirulence even though the double mutant exhibited the SpeB hyperexpression phenotype of SpxA2^−^.

**FIG 7:**
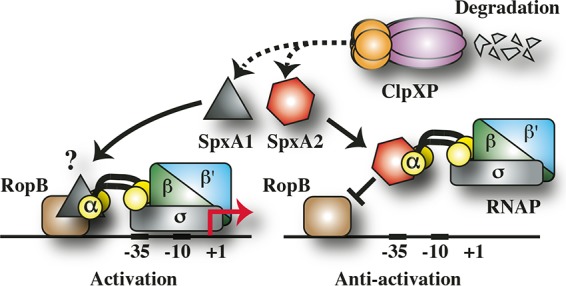
Model of regulation of SpeB. The data presented here and the known properties of SpxA1 and SpxA2 are consistent with a model of SpeB regulation in which SpxA1 and SpxA2 are in competition for binding to the C-terminal domain of the α subunit (αCTD) of RNAP (which also consists of the subunits β, β′, and σ). Bound SpxA2 inhibits the ability of RopB, an essential activator of *speB* transcription, from recruiting RNAP to the *speB* promoter (antiactivation). In contrast, RopB can recruit RNAP when αCTD is occupied by SpxA1 (activation), leading to expression of *speB* (shown by the red arrow at +1). Turnover of SpxA1 and SpxA2 (shown by the dashed line) is via the ClpXP proteasome (degradation). Gateways for regulatory input to change the steady state are numerous and may involve modulation of the relative concentrations of SpxA1 and SpxA2 or differential modification of their binding affinities for αCTD and ClpX. The question mark indicates that it has not been established whether SpxA1 interacts directly with RopB or that its positive influence is derived solely from its ability to displace SpxA2 from αCTD. Regardless, the antagonistic activities of SpxA1 and SpxA2 allow the interrogation of multiple regulatory inputs for fine-tuning of SpeB expression.

In addition to regulation of distinct gene subsets, the finding that SpxA1 and SpxA2 have opposing effects on infection in soft tissue suggests that they act coordinately to regulate virulence. Both paralogs are highly homologous and share a conserved glycine residue (see Fig. S2) that has been shown to be important for binding to αCTD of RNAP (20). This suggests a fine-tuning mechanism involving competition between SpxA1 and SpxA2 for binding to αCTD. In this model, the loading of a specific SpxA paralog into RNAP will alter how subsets of virulence gene transcription regulators interact with RNAP. Furthermore, the two paralogs will act antagonistically because the presence of one will exclude the other from binding to RNAP. For SpeB, SpxA2 acts in a negative fashion, suggesting that it blocks the ability of RopB, a member of the Rgg family of DNA-binding proteins (27), to recruit RNAP to the *speB* promoter (antiactivation, Fig. 7). In contrast, RopB can likely recruit the SpxA1-RNAP complex to activate *speB* transcription (activation, Fig. 7). Adaptation results from input into the network that alters the steady state by changing the relative concentrations of the active form of any of the components through differential changes in the expression of component genes, through modifications of individual components that alter binding activity, or through differential modulation of ClpXP-mediated turnover of SpxA1 versus SpxA2 (degradation, Fig. 7). This model explains how the loss of ClpX results in repression of *speB* transcription (by overaccumulation of SpxA2) that can be suppressed by overexpression of *ropB* or by the subsequent loss of SpxA2. Refinement of this model will require uncovering of the molecular details of the various binding interactions and the regulatory inputs that modulate the steady state in response to specific stimuli.

SpxA2 was also involved in the regulation of PB^r^, as higher levels of SpxA2 in the ClpX^−^ mutant resulted in PB^r^, subsequent loss of SpxA2 suppressed PB^r^ in a double mutant, and a single SpxA2^−^ mutant became more polymyxin B sensitive than the WT. The single SpxA2^−^ mutant also became sensitive to numerous other stresses that are thought to be characteristic of the *in vivo* environment. Remarkably, the highly stress-sensitive SpxA2^−^ mutant was hypervirulent as defined by more extensive tissue damage and an ability to achieve a greater tissue burden of bacteria than the WT. In this regard, it should be noted that the SpxA1^−^ mutant, while attenuated versus the WT, was still able to cause tissue damage and grow in soft tissue, despite its inability to grow aerobically. Given the intensity of the host’s inflammatory response to *S. pyogenes*, it is highly unlikely that these stresses are missing *in vivo*, raising the interesting question of how the stress-sensitive SpxA mutants can proliferate. Infection by *S. pyogenes* can result in extensive remodeling of the surrounding soft tissue environment (4), suggesting that these alterations create a protective niche that can insulate the bacteria from excessive stress. In this case, it would be predicted that the SpxA2^−^ mutant will be attenuated in models of disseminated infection that do not involve extensive remodeling. Regardless of the mechanism, these data do show that sensitivity to stress *in vitro* is not necessarily predictive of a loss of virulence *in vivo*. To the best of our knowledge, this is the first example of an SpxA^−^ mutant that is hypervirulent, as SpxA^−^ mutants of *S. mutans* (29), *S. sanguinis* (23), and *Enterococcus faecalis* (28) have been shown to be attenuated in various animal models.

In summary, the data presented in this report show that SpxA1 and SpxA2 work antagonistically to regulate *S. pyogenes* virulence. The fact that the two proteins likely compete for occupancy on RNAP suggests that they act coordinately to fine-tune virulence in concert with various mechanisms of signal transduction, including the ExPortal and regulated proteolysis via ClpXP, to bind to RNAP and regulate the expression of different subsets of the *S. pyogenes* transcriptome. In further work, it will be important to test whether SpxA2 mutation can result in hypervirulence in diverse *S. pyogenes* strains. In this regard, a genome-wide transposon sequencing analysis of fitness during *in vitro* culture has shown that transposon insertion in SpxA1 had no effect on fitness in an M49 strain and an M1T1 isolate that has been associated with more virulent infections (35). In contrast, insertion in SpxA2 did not affect fitness in the M49 strain but did affect fitness in the M1T1 strain (35). This suggests that heterogeneity in the SpxA signaling network exists. Further characterization of these SpxA networks will provide insight into how stress is interrogated by *S. pyogenes* for time- and compartment-specific regulation of virulence factor expression.

## MATERIALS AND METHODS

### Strains, media, and growth conditions.

Routine molecular cloning and plasmid propagation used *Escherichia coli* DH5α, which was cultured in Luria-Bertani medium at 37°C. Experiments with *S. pyogenes* used strain HSC5 (36, 37) and mutant derivatives of this strain listed in Table S1. Media and culture conditions for anaerobic growth have been described in detail elsewhere (7). When appropriate, antibiotics were added at the following concentrations: erythromycin, 1 μg ml^−1^ for *S. pyogenes* and 500 μg ml^−1^ for *E. coli*; kanamycin, 500 μg ml^−1^ for *S. pyogenes* and 50 μg ml^−1^ for *E. coli*.

### DNA techniques.

Plasmid DNA was isolated via standard techniques and used to transform *S. pyogenes* or *E. coli* as previously described (38). In-frame deletion mutations in chromosomal loci were generated by standard methods with the temperature-sensitive shuttle vectors pJRS233 (39) and pGCP213 (40) to construct the mutagenic plasmids listed in Table S2. Each deletion allele was generated by PCR with oligonucleotide primers (IDT, Coralville, IA) listed in Table S3 by overlap extension PCR (41). Selected mutants were constructed by an integration strategy (7) with the suicide vector pSPC18 (42) to generate the integrational plasmids described in Table S2. Modified alleles were inserted directly into plasmids at the M13F and M13R universal primer-binding sites by the overlap extension PCR cloning method described in detail elsewhere (43). Complementation plasmids were generated with the shuttle vector pABG5 (44) to insert open reading frames along with upstream ribosome-binding sites downstream of the *rofA* promoter by standard techniques as previously described (45). For ectopic chromosomal complementation, *clpX* was inserted into the chromosome directly downstream of *guaB*, generating a polycistron driven by the *guaB* promoter (see Text S1 for details). The fidelity of all molecular constructs and mutated chromosomal loci was confirmed by PCR and determination of DNA sequences (Genewiz, South Plainfield, NJ) with appropriate primers. Selected mutants were subjected to whole-genome sequencing and independently rederived to rule out the contributions of any adventitious mutations to the phenotypes reported.

10.1128/mBio.00288-17.7 TABLE S2  Plasmids used in this study. Download TABLE S2, PDF file, 0.1 MB. Copyright © 2017 Port et al. 2017 Port et al. This content is distributed under the terms of the Creative Commons Attribution 4.0 International license.

10.1128/mBio.00288-17.8 TABLE S3  Primers used in this study. Download TABLE S3, PDF file, 0.1 MB. Copyright © 2017 Port et al. 2017 Port et al. This content is distributed under the terms of the Creative Commons Attribution 4.0 International license.

### Generation of SpxA2 deletion mutants.

SpxA1 deletion mutants and deletion of SpxA2 in a ClpX^−^ background were readily obtainable; however, it was not possible to directly obtain certain SpxA2 deletion mutants. An alternative approach to restore a WT copy of *clpX* into the original genomic locus in ClpX^−^ SpxA2^−^ and ClpX^−^ SpxA1^−^ SpxA2^−^ mutants succeeded in generating the SpxA2^−^ and SpxA1^−^ SpxA2^−^ mutants, respectively (see Table S1). These mutants had growth defects (Table S4), which likely complicated the recovery of mutants by the original strategy.

10.1128/mBio.00288-17.9 TABLE S4  Growth rates and yields of various *S. pyogenes* mutants. Download TABLE S4, PDF file, 0.1 MB. Copyright © 2017 Port et al. 2017 Port et al. This content is distributed under the terms of the Creative Commons Attribution 4.0 International license.

### Analysis of SpeB expression.

The ability of various strains to express active SpeB protease activity was assessed on protease indicator plates (solidified C medium supplemented with 2% milk) (27) by plating 10-fold serial dilutions and examining the resulting zones of clearance following 16 h of incubation (7) or in culture supernatants with a fluorescein isothiocyanate-casein cleavage assay as previously described (27), with minor modifications to adapt to a 96-well format (Text S1 for details). The ability to secrete and process the SpeB zymogen was assessed by Western blotting analyses of culture supernatants with an anti-SpeB rabbit serum (catalog number ab53403; Abcam, Inc., Cambridge, MA) as previously described (7). Samples were normalized to the culture optical density at 600 nm (OD_600_) at the time of harvest. The data presented are representative of at least three independent experiments.

### Analysis of transcripts.

The transcript abundance of selected genes was analyzed by real-time RT-PCR as previously described (7). Briefly, overnight cultures were diluted 1:25 or 1:10 in fresh C medium and RNA extracted from samples harvested at mid-logarithmic phase (50% of final OD_600_), late logarithmic phase (75% of final OD_600_), stationary phase (100% of final OD_600_), or 2 h after stationary phase onset with a commercially available kit in accordance with the manufacturer’s instructions (Direct-Zol RNA MiniPrep, catalog number R2052; Zymo Research, Irvine, CA), with the following minor modifications. A 10-ml culture volume was subjected to centrifugation at 6,000 × *g* for 5 min, and the cell pellet was frozen at −80°C. The cell pellet was then thawed, resuspended in 700 μl of Trizol, and subjected to bead beating with a FastPrep-24 homogenizer (MP Biomedicals, Santa Ana, CA) for 2 pulses at 6.5 m/s for 45 s, separated by a 5-min resting period, at 4°C. Lysates were subjected to centrifugation at 500 × *g* for 30 s, mixed with 100% ethanol, and loaded into RNA capture columns as directed by the manufacturer. Selected genes were analyzed by real-time RT-PCR with the oligonucleotide primers listed in Table S3. Relative *speB* transcript levels are displayed as −Δ*C*_*T*_, normalized to *recA* for each strain and time point, while relative transcript levels of *slo* are displayed as –ΔΔ*C*_*T*_, normalized to *recA* and relative to the WT as previously described (7). The data shown are the mean and the standard error of the mean derived from duplicate determinations of samples from at least two independent experiments.

### Determination of PB^r^, growth rate, and maximum growth temperature.

PB^r^ was quantitated by an efficiency-of-plating assay on polymyxin B-supplemented medium as previously described (7). Growth rates and yields were determined by monitoring growth in C medium and calculated as described previously (7). The maximum growth temperature was determined with C medium and incubating multiple 250-μl cultures of each strain indicated in a thermocycler (Mastercycler pro S; Eppendorf, Hauppauge, NY) set at a constant temperature with a 1°C gradient spread over 12 adjacent wells. The maximum growth temperature (*T*_max_) was defined as the highest temperature with visible growth where at least three sequential wells at lower temperatures had visible growth and three sequential wells at higher temperatures had no visible growth. The data presented are the mean and standard error derived from at least five independent experiments.

### Aerobic growth.

The ability of strains to grow aerobically was tested as follows. Strains were cultured overnight in 10 ml of C medium in sealed culture tubes at 37°C under static conditions. Cultures were then back diluted 1:1,000 into 5 ml of fresh medium in loosely capped glass test tubes (18 by 150 mm) and cultured at 37°C either in a roller drum spinning at 86 rpm (aerobic) or in a sealed jar with a commercial gas generator (anaerobic; catalog number B260001; Fisher, Waltham, MA). A 500-μl sample was removed every 24 h over the course of 2 days to measure the OD_600_ and determine the number of CFU ml^−1^ by spotting serial dilutions onto solid medium that was incubated anaerobically. The data presented are the mean and standard error derived from at least three independent experiments.

### Oxidative stress resistance.

The oxidizing agents diamide (catalog number D3648; Sigma, St. Louis, MO) and paraquat (methyl viologen, catalog number 856177; Sigma) were added to C medium as follows. Diamide was added to achieve final concentrations ranging from 0 to 275 μM in 25 μM stepwise increments. Paraquat was added in either a small-dose series (0 to 1 mM in 0.1 mM stepwise increments) or a large dose series (0 to 10 mM in 1 mM stepwise increments). For analysis, aliquots of supplemented test medium (200 μl) were added to individual wells of a 96-well plate (catalog number 3370; Corning, Corning, NY) and inoculated from frozen glycerol stocks. Plates were covered with loosely fitting lids and wrapped with Saran wrap to avoid evaporation and incubated for 24 h at 37°C under constant rotation at 200 rpm. Growth yield was monitored by measuring the OD_600_ with a Tecan Infinite M200 Pro plate reader. The MIC was defined as the lowest concentration of oxidizing agent that resulted in <10% of the final growth yield relative to control medium lacking oxidizing agent (OD_600-test_/OD_600-control_ = <10%). The data presented are the mean and standard error from at least five independent experiments.

### Subcutaneous ulcer model of infection.

Infection of mice followed a well-established protocol. Briefly, 6- to 8-week-old female SKH1 mice (Charles River Laboratories, Inc., Wilmington, MA) were injected with approximately 10^7^ CFU of the bacterial strains indicated by a previously described method (46, 47). Following infection, the resulting ulcers were examined over the course of 3 days and documented by digital photography. The areas of the resulting irregular lesions were calculated from captured images with ImageJ (http://imagej.nih.gov/ij/). To quantify bacterial CFU counts at the lesion sites, mice were sacrificed at 72 h postinfection and the infected mouse tissue samples were excised and placed into 2-ml screw-cap tubes (catalogue number 02-707-355; Fisher) along with two 5-mm stainless steel beads (catalog number 69989; Qiagen, Valencia, CA) and 1 ml of pH 11 water (48). Tissue samples were homogenized with a FastPrep-24 homogenizer (MP Biomedical) at speed 6.0 m/s for two bursts of 45 s each, separated by a 5-min resting period, at 4°C. Aliquots were then serially diluted and spotted onto solid medium, which was incubated at 37°C under anaerobic conditions. To quantify SpeB expression, homogenized samples were subjected to centrifugation at 500 × *g* for 5 min at 4°C, and 200 μl of supernatant was collected and then subjected to an additional round of centrifugation at 6,000 × *g* for 5 min at 4°C. Aliquots were normalized to bacterial CFU counts and assessed by Western blot analysis (see above). The data presented are pooled from two independent experiments with 10 mice per experimental group.

### Ethics statement.

This study was carried out in accordance with the Public Health Service Policy on Humane Care and Use of Laboratory Animals and AAALAC accreditation guidelines. The protocols were approved by Washington University in St. Louis’ Animal Studies Committee (Animal Welfare Assurance number A-3381-01 and protocol number 20140061).

### Statistical analyses.

For *in vitro* assays, differences between mean values were tested for significance with the Dunnett test. Differences between WT and mutant strains in mouse lesion size and CFU counts were tested for significance with the Mann-Whitney test. Test statistics were calculated with the InStat module of GraphPad (version 3.06; GraphPad Software, Inc., La Jolla, CA). For all tests, the null hypothesis was rejected for *P* values of >0.05.
