# Supplementary figures and images for: SpxA1 and SpxA2 Act Coordinately To Fine-Tune Stress Responses and Virulence in Streptococcus pyogenes
Source: mBio. 2017 Mar 28;8(2):e00288-17. doi: 10.1128/mBio.00288-17 (PMC5371413; doi:10.1128/mBio.00288-17)

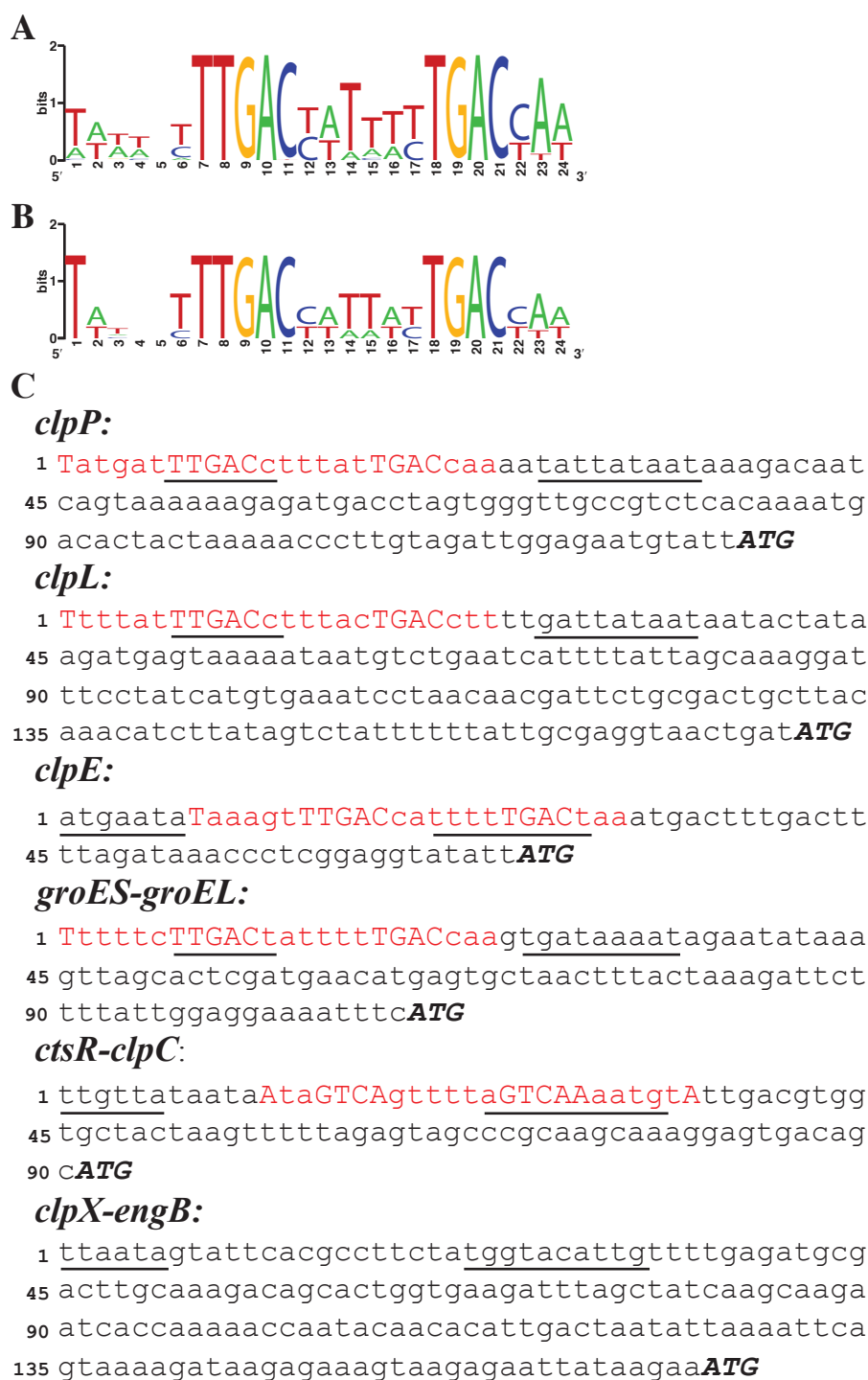

**Figure S1**

Supplement: FIG S1 [file mbo002173246sf1.pdf]

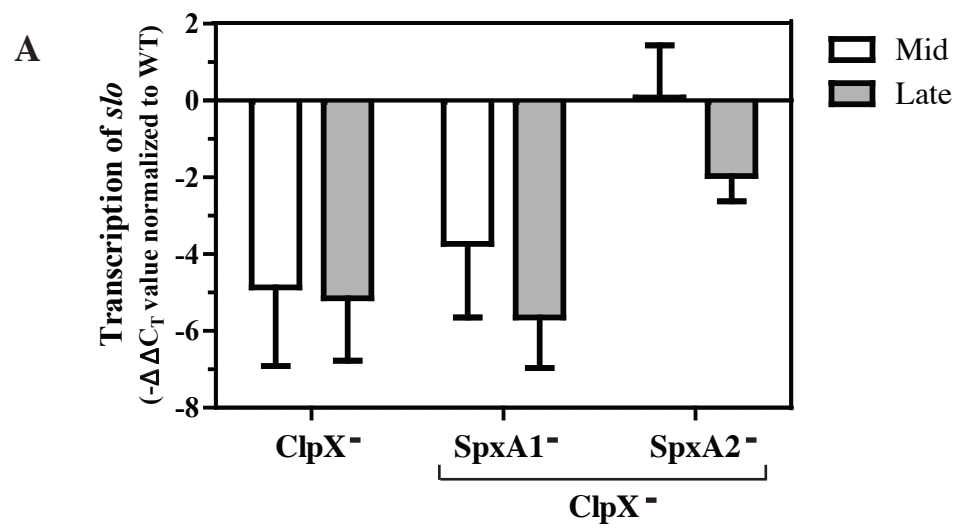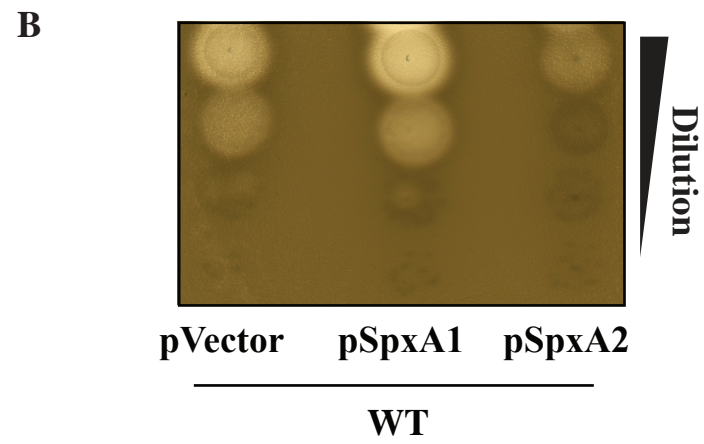

**Figure S3**

Supplement: FIG S3 [file mbo002173246sf3.pdf]

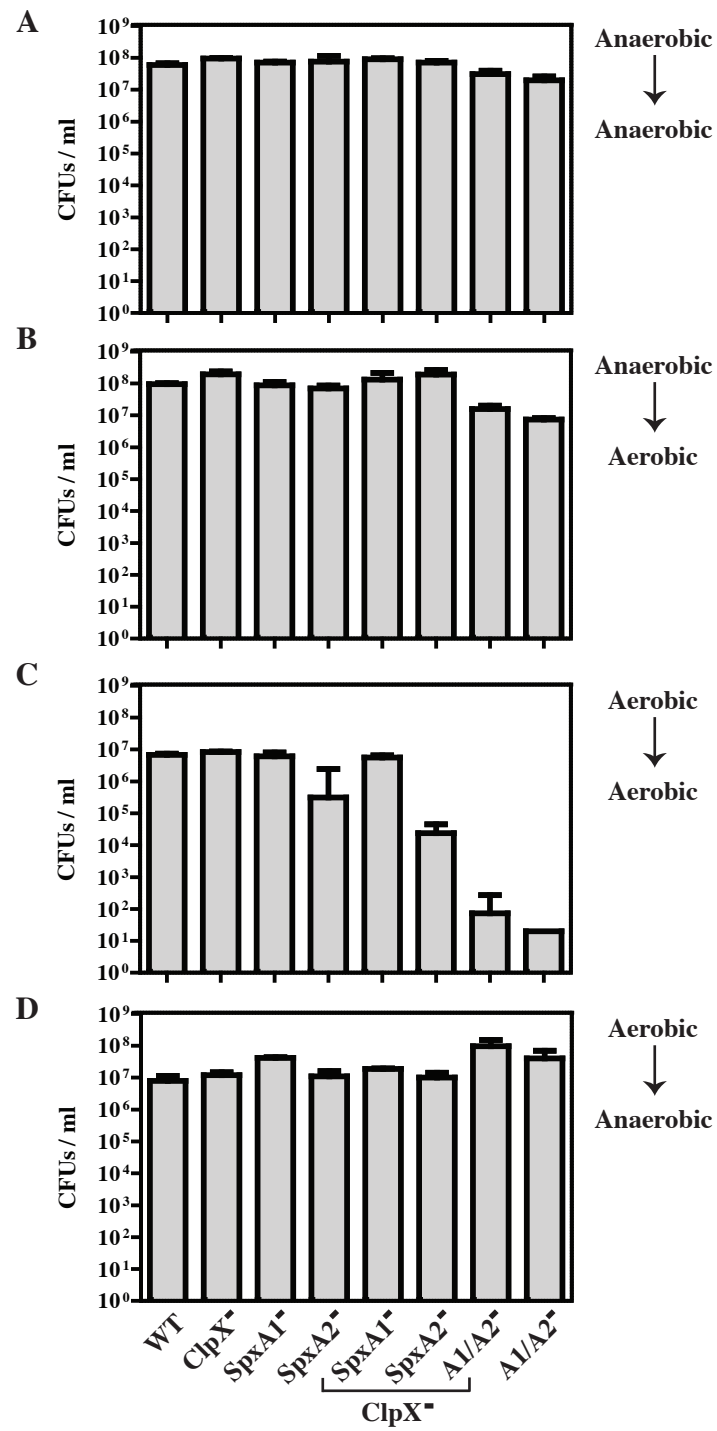

**Figure S4**

Supplement: FIG S4 [file mbo002173246sf4.pdf]

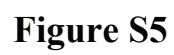

### Figure S5

Supplement: FIG S5 [file mbo002173246sf5.pdf]
