# Supplementary material for: SpxA1 and SpxA2 Act Coordinately To Fine-Tune Stress Responses and Virulence in Streptococcus pyogenes
Source: mBio. 2017 Mar 28;8(2):e00288-17. doi: 10.1128/mBio.00288-17 (PMC5371413; doi:10.1128/mBio.00288-17)
Supplement: TABLE S1 [file mbo002173246st1.pdf]

Table S1. Bacterial strains containing engineered and complemented mutations used in this study

Strains (alternate name)	Relevant Genotype	Mutated Loci[a]	Plasmid (alternate name)[b]	Parental strain[c]	Comment[d]	Reference
**WT or deletion mutants**						
HSC5	wild type (WT)	NA				(1)
GCP688	ClpX$^-$	03620	pGCP666	HSC5		(2)
GCP1245 (ZC611)	SpxA1$^-$	04745	pGCP1289 (pZC165)	HSC5		This study
GCP1033	SpxA2$^-$	08945	pGCP1001	GCP726		This study
GCP711	ClpX$^-$/SpxA1$^-$	03620, 04745	pGCP1289 (pZC165)	GCP688		This study
GCP726	ClpX$^-$/SpxA2$^-$	03620, 08945	pGCP1290 (pZC192)	GCP688		This study
GCP729	ClpX$^-$/SpxA1$^-$/SpxA2$^-$	03620, 04745, 08945	pGCP1290 (pZC192)	GCP711		This study
GCP1072	SpxA1$^-$/SpxA2$^-$	04745, 08945	pGCP1001	GCP729		This study
GCP1255	SpxA2$^-$/SpeB$^-$	08945, 08645	pGCP485 (pCK365)	GCP1033		This study
GCP538	SpeB$^-$	08645	pGCP485 (pCK365)	HSC5		This study
JWR100 (GCP057)	SpeB$_{C192S}$	08645			Enzymatically inactive allele of SpeB	(3)
MNN100 (GCP543)	RopB$^-$	08655				(4)
**Insertional disruption or antibiotic cassette swap mutants**						
GCP652	ΩClpP	01780	pGCP647	HSC5	SpcR, plasmid insertion within *clpP*	This study
GCP653	ΩClpL	03635	pGCP648	HSC5	SpcR, plasmid insertion within *clpL*	This study
GCP654	ΩClpE	06190	pGCP649	HSC5	SpcR, plasmid insertion within *clpE*	This study
GCP655	ΩCtsR-ClpC	08770	pGCP651	HSC5	SpcR, plasmid insertion within *ctsR*, polar on *clpC*	This study
GCP656	ΩClpC	08765	pGCP650	HSC5	SpcR, plasmid insertion within *clpC*	This study
GCP1300	ClpP$^-$::*aad9*	01780	pWAR251 (pGCP1291)	HSC5	SpcR, allelic replacement of *clpP* with *aad9*	This study
**Complemented mutants**						
GCP017	WT + pVector	NA	pABG5 (pVector)	HSC5	KanR, multicopy plasmid	This study
GCP929	WT + pRopB-HA	NA	pJL60 (pGCP694)	HSC5	KanR, RopB-HA expressed on multicopy plasmid	This study
GCP790	ClpX$^-$ + pVector	03620	pABG5 (pVector)	GCP688	KanR, multicopy plasmid	This study
GCP695	ClpX$^-$ + pClpX	03620	pGCP893 (pZC154)	GCP688	KanR, ClpX expressed on multicopy plasmid	This study
GCP705	ClpX$^-$ + ClpX^R	03620	pGCP610	GCP688	ClpX exressed on chromosome downstream of *guaB*	This study
GCP696	ClpX$^-$ + pRopB-HA	03620	pJL60 (pGCP694)	GCP688	KanR, RopB-HA expressed on multicopy plasmid	This study
GCP931	RopB$^-$ + pVector	08655	pABG5 (pVector)	MNN100 (GCP543)	KanR, multicopy plasmid	This study
JL139 (GCP930)	RopB$^-$ + pRopB-HA	08655	pJL60 (pGCP694)	MNN100 (GCP543)	KanR, RopB-HA expressed on multicopy plasmid	(5)
ZC572	WT + pSpxA1-6xHis	NA	pZC169	HSC5	KanR, SpxA1-6xHis expressed on multicopy plasmid	This study
ZC573	WT + pSpxA2-6xHis	NA	pZC170	HSC5	KanR, SpxA2-6xHis expressed on multicopy plasmid	This study

[a]Loci are based on the genome of HSC5 (1) and follow the format L897_xxxxx, where xxxxx is the number listed in the Table. NA, not applicable.

[b]Mutagenic plasmid (Table S2) used to delete or disrupt endogenous gene(s) in HSC5. Empty vector and complementation plasmids (pVector, pClpX, pRopB-HA; Table S2) used to restore or overexpress select genes. See the Experimental Procedures for details.

[c]Parental strain used to derive described mutant.

[d]Antibiotic resistances are abbreviated as follows: spectinomycin (SpcR), kanamycin (KanR).

REFERENCES

1. Port GC, Paluscio E, Caparon MG (2013) Complete Genome Sequence of emm Type 14 *Streptococcus pyogenes* Strain HSC5. Genome Announc 1: 612-613.
2. Port GC, Vega LE, Nylander AB, Caparon MG (2014) *Streptococcus pyogenes* polymyxin B-resistant mutants display enhanced ExPortal integrity. J Bacteriol 196: 2563-2577.
3. Loughman JA, Caparon M (2006) Regulation of SpeB in *Streptococcus pyogenes* by pH and NaCl: a model for in vivo gene expression. J Bacteriol 188: 399-408.
4. Neely MN, Lyon WR, Runft DL, Caparon M (2003) Role of RopB in growth phase expression of the SpeB cysteine protease of *Streptococcus pyogenes*. J Bacteriol 185: 5166-74.
5. Loughman JA, Caparon M (2007) Contribution of invariant residues to the function of Rgg family transcription regulators. J Bacteriol 189: 650-655.
