# Supplementary material for: SpxA1 and SpxA2 Act Coordinately To Fine-Tune Stress Responses and Virulence in Streptococcus pyogenes
Source: mBio. 2017 Mar 28;8(2):e00288-17. doi: 10.1128/mBio.00288-17 (PMC5371413; doi:10.1128/mBio.00288-17)
Supplement: FIG S2 [file mbo002173246sf2.pdf]

```
ORF   Locus     (Prefix)

SpxA1                        *: *: ***********:***  *.: : *:**::. :.  ::  **  **:***::******* :*** ::::.: :. *  :: . * ******: * *::*:*:****** ** *  *  :: .:
SpxA1 L897_04745 (spyh) MVTLFLSPSCTSCRKARAWLVKHEVDFQEHNIITSPLSRDELMSILSFTENGTEDIISTRSKVFQKLDVDVEELSISDLIDLIAKNPSLLRRPIIMDQKRMQIGFNEDEIRAFLSRDYRKQELRQATIKAEIEG---
SpxA1 SPy_1249   (spy)  MVTLFLSPSCTSCRKARAWLVKHEVDFQEHNIITSPLSRDELMSILSFTENGTEDIISTRSKVFQKLDIDVEELSISDLIDLIAKNPSLLRRPIIMDQKRMQIGFNEDEIRAFLSRDYRKQELRQATIKAEIEG---
SpxA  SMU_1142c  (smu)  MVTLFLSPSCTSCRKARAWLNRHDVVFQEHNIMTSPLSRDELLKILSYTENGTEDIISTRSKVFQKLDIDVDELSVSELINLISKNPSLLRRPIIMDNKRMQIGFNEDEIRAFLPRDYRKQELRQATIRAEVEGEDD
SpxA1 SP_1405    (spn)  MITLFLSPSCTSCRKAKAWLEKHKVPFVEHNIMTSPLTRKELQHILSLTENGTDDIISTRSKIFQKLNIDVESISVSELLHLIEQYPSLLRRPIIIDAKRMQIGFNEDEIRAFLPRSYRKQELKEARMRAGIS----
SpxA1 SSA_0937   (ssa)  MITLFLSPSCTSCRKARAWLLNHEVPFQEHNIMTSPLSAPELQHILSLTENGTDDIISTRSKIFQKLDLDVEDLSISTLIQLIEENPSLLRRPIILDGKRMQIGFNEDEIRAFLPRSYRKEELRSATMRADIQ----
SpxB  L132585    (lla)  MIDLYLSPSCTSCRKARAWLQSHKVPFVEHNILTQPMTTNDLRHILTKTENGTEDIISTRSKVFQKLAVDVDNLTINELLDLVTEFPNLLRRPIITDSKHLQIGFNEDEIRAFLPREYRRAEMLSTID---------
SpxA  EF2678     (efa)  MLTLYTSPSCTSCRKARAWLQEHEIPFKERNIFSEPLNIEELKAILIMTEDGTEEIISTRSKVFQKLNMDLDELPLQDLLELVQENPGLLRRPIMIDEKRLQVGFNEDEIRRFLPRDVRQLELRQAQLMAGL-----
SpxA  SA0856     (sau)  MVTLFTSPSCTSCRKAKAWLQEHDIPYTERNIFSEHLTIDEIKQILKMTEDGTDEIISTRSKTYQKLNVDIDSLPLQDLYSIIQDNPGLLRRPIILDNKRLQVGYNEDEIRRFLPRKVRTFQLQEAQRMVD------
SpxA  BSU11500   (bsu)  MVTLYTSPSCTSCRKARAWLEEHEIPFVERNIFSEPLSIDEIKQILRMTEDGTDEIISTRSKVFQKLNVNVESMPLQDLYRLINEHPGLLRRPIIIDEKRLQVGYNEDEIRRFLPRKVRSFQLREAQRLAN------
SpxA  lmo2191    (lmo)  MVTLYTSPSCTSCRKARAWLEEHDIPYKERNIFSEPLSLDEIKEILRMTEDGTDEIISTRSKTFQKLNVDLDSLPLQQLFELIQKNPGLLRRPIIIDEKRLQVGYNEDEIRRFLPRRVRTYQLREAQKMVN------

SpxA2                        **.***  ************. *:: ::*:**  : ::  *:  ** * ::*: .::**:**:.* *  :::::*:.:.:.:* :.*:*:: **::*:***::**:*::*******::* :**  ****:*:
SpxA2 L897_08945 (spyh) MIKIYTISSCTSCKKAKTWLNAHKLAYKEQNLGKEPLTKEEILAILSKTENGVESIVSSKNRYAKALDCDIEELSVSEVIDLIQDNPRILKSPILIDDKRLQVGYKEDDIRAFLPRSIRNIENTEARLRAAL-----
SpxA2 SPy_2115   (spy)  MIKIYTISSCTSCKKAKTWLNAHKLAYKEQNLGKEPLTKEEILAILSKTENGVESIVSSKNRYAKALDCDIEELSVSEVIDLIQDNPRILKSPILIDDKRLQVGYKEDDIRAFLPRSIRNIENTEARLRAAL-----
SpxB  SMU_2084c  (smu)  MIKIYTISSCTSCKKAKTWLNAHQLPYKEQNLAKDPLSKEEILNILSKTENGIESIVSSKNRYAKALHCNIDDLSVNEVIDLIQENPRILKSPILIDDKRLQIGYKEDDIRAFLPRSIRNVENTAARLRAAL-----
SpxA2 SP_0189    (spn)  MIKIYTVSSCTSCKKAKTWLNAHQLSYKEQNLGKEGITREELLDILTKTDNGIASIVSSKNRYAKALGVDIEDLSVNEVLNLIMETPRILKSPILVDEKRLQVGYKEDDIRAFLPRSVRNVENAEARLRAAL-----
SpxA2 SSA2244    (ssa)  MITIYTVSSCTSCKKAKTWLNAHQLTYKEQNLGKEGITKEELLDILTKTENGIASIVSSKNRYAKGLGVDIEELSVNEVLDIIMETPRILKSPILVDDKRLQVGYKEDDIRAFLPRSVRNVENAEARLRAAL-----
TrmA  L73853     (lla)  MITIYTAPSCTSCKKAKTWLSYHHIPFNERNLIADPLSTTEISQILQKCDDGVEGLISSRNRFVKTLGVDFEDISLSQAIKIISENPQIMRRPIIMDEKRLHVGYNEEEIRAFLPRTVRVLENGGARLRSAI-----
                        *: ::   *****:**::**  *.: : *:*:  . :.  ::  **   ::*   ::*::.:  : *  :.:.: :.    :: . * ::: **: * *::::*::*::** ** *  *  :   :
                                 CxxC                                        G
```

Figure S2
