# Supplementary material for: SpxA1 and SpxA2 Act Coordinately To Fine-Tune Stress Responses and Virulence in Streptococcus pyogenes
Source: mBio. 2017 Mar 28;8(2):e00288-17. doi: 10.1128/mBio.00288-17 (PMC5371413; doi:10.1128/mBio.00288-17)
Supplement: TEXT S1 [file mbo002173246s1.docx]

**SUPPLEMENTARY METHODS**

**Binding site analyses.** The consensus CtsR binding sequence was derived from 68 putative binding sites from a variety of streptococcal and lactococcal species using the online software RegPrecise (<http://regprecise.lbl.gov>) (1). The consensus CtsR binding sequences were generated using the online software WebLogo (<http://weblogo.berkeley.edu/>) (2) by analyzing all 68 putative sites as originally curated in RegPrecise with the exception that *ctsR*, *yffA* (*Lactococcus lactis*) and *clpL* (*S. thermophilus*) binding sites were inverted to analyze their reverse complement sequences. Sequences used to derive the consensus *S. pyogenes* CtsR binding site were derived from the HSC5 genome (3), from the following genes and loci: *clpP*, L897_01780; *clpL*, L897_03635; *clpE*, L897_06190;, *groS*, L897_08760; *ctsR*, L897_08770; *clpX*, L897_03620. Putative -35 and -10 promoter elements were identified using the online software BProm (Softberry, Inc, Mount Kisco, NY) (4).

Primary amino acid sequences were derived from the Kegg Genome Database (<http://www.genome.jp/kegg/genes.html>) and abbreviations are as follows in the format: locus-prefix (species, strain); spyh (*S. pyogenes*, HSC5); spy (*S. pyogenes*, SF370); smu (*S. mutans*, UA159); spn (*S. pneumoniae*, TIGR4); ssa (*S. sanguinis*, SK36); efa (*Enterococcus faecalis*, V583); lla (*Lactococcus lactis*, Ll1403); sau (*Staphylococcus aureus*, N315); bsu (*Bacillus subtilis*, 168); lmo (*Listeria monocytogenes*, EGD-e). Amino acid alignments were performed using the online software Clustal Omega (<http://www.ebi.ac.uk/Tools/msa/clustalo/>).

**Ectopic chromosomal complementation.** Ectopic chromosomal complementation of *clpX* using pGCP610 was performed as follows; 500bp upstream and downstream (1kb total) surrounding the stop codon of *guaB* was cloned into pJRS233 generating pGCP140b. *sfGFP* was then inserted at a point 10bp downstream from the *guaB* stop codon, but before the transcriptional terminator generating pGCP189. *clpX* was then amplified and used to replace sfGFP generating pGCP610. Finally, *clpX* was inserted into the chromosome directly downstream of *guaB* generating a polycistron driven by the *guaB* promoter using homologous recombination.

**Growth rate and yield analysis.** Bacterial growth was monitored in a 96-well plate as previously described (5). Briefly, overnight cultures grown in C-medium were back diluted 1:50 into fresh C-medium or ThyB and 200 μl aliquots were placed in triplicate into a clear flat-bottom 96-well plate, covered with optical tape, and incubated at 37C. Growth was monitored every 5 minutes for 24 hours in a Tecan-infinite M200 Pro plate reader.

**Analysis of SpeB expression.** The ability of various strains to express SpeB protease activity was assessed in culture supernatants using a FITC-casein cleavage assay as described (6), with minor modifications to adapt to a 96-well format. Briefly, overnight cultures in C medium grown from single isolated colonies were back-diluted 1:100 into fresh C medium and grown for 20-24 hrs at 37^o^C. Density of overnight cultures was measured by OD_600_ and the remaining culture was subjected to centrifugation at 10,000 x g for 5 min. Supernatants were collected and immediately frozen at -20ºC. To quantify protease activity, supernatants were thawed and normalized by OD_600_ relative to WT by dilution with fresh C medium and 100 μl of normalized supernatant was placed into individual wells of a black flat bottom 96-well plate (catalog #7605; Fisher) in duplicate. 100 μl of 400 μg ml^-1^ fluorescein isothiocyanate (FITC)-casein (catalog #C3777; Sigma) suspended in SpeB assay buffer (0.1 M sodium phosphate [pH 6.6], 1mM EDTA, 0.1mM DTT) was then added to each well and immediately placed in a Tecan Infinite M200 Pro plate reader for excitation 490 nm emission 525 nm measurements at time 0 and after 5 minutes incubation at 37ºC to measure protease-dependent release of fluorescein. Differences in emission at 5 minutes relative to time 0 were compared to wild type which was set at 100%. Cysteine protease inhibitor E64 (28 μM, Sigma) was added to selected samples before overnight growth to confirm protease activity was dependent upon SpeB. Values reported are the means and standard error of the means from at least three independent experiments. Data presented are representative of at least 3 independent experiments.

**SUPPLEMENTARY METHODS REFERENCES**

1. Novichkov PS, Laikova ON, Novichkova ES, Gelfand MS, Arkin AP, Dubchak I, Rodionov DA. 2010. RegPrecise: a database of curated genomic inferences of transcriptional regulatory interactions in prokaryotes. Nucleic Acids Res 38:D111-8.

2. Crooks GE, Hon G, Chandonia JM, Brenner SE. 2004. WebLogo: a sequence logo generator. Genome Res 14:1188-90.

3. Port GC, Paluscio E, Caparon MG. 2013. Complete Genome Sequence of emm Type 14 *Streptococcus pyogenes* Strain HSC5. Genome Announc 1:612-13.

4. Solovyev V, Salamov A. 2011. Automatic annotation of microbial genomes and metagenomic sequences, p 61-78. *In* Li RW (ed), In Metagenomics and its Applications in Agriculture, Biomedicine and Environmental Studies. Nova Science Publishers.

5. Port GC, Vega LA, Nylander AB, Caparon MG. 2014. *Streptococcus pyogenes* polymyxin B-resistant mutants display enhanced ExPortal integrity. J Bacteriol 196:2563-77.

6. Lyon WR, Gibson CM, Caparon MG. 1998. A role for trigger factor and an rgg-like regulator in the transcription, secretion and processing of the cysteine proteinase of *Streptococcus pyogenes*. EMBO J 17:6263-75.

**SUPPLEMENTARY FIGURES AND TABLES**

**Figure S1. Predicted CtsR binding sites in *S. pyogenes***. **(A)** CtsR consensus binding sequence derived from 68 predicted CtsR binding sites from a variety of streptococcal and lactococcal species. **(B)** CtsR consensus binding sequence derived from five predicted *S. pyogenes* CtsR binding sites. **(C)** 5’ promoter regions of indicated genes are displayed as sense strands with the exception of *ctsR*, which is displayed as antisense. Promoter regions of indicated genes are marked to include putative -35 and -10 transcriptional sites (underlined), CtsR binding sites (red), invariant residues (uppercase) and start ATG codons (italicized). Polycistronic genes are indicated with hyphens.

**Figure S2. Alignment of SpxA1 and SpxA2 amino acid sequences from *S. pyogenes* and other Gram-positive bacteria**. Sequence alignment of SpxA1 and SpxA2 from *S. pyogenes* (spy and spyh), *S. mutans* (smu) (1), *S. pneumoniae* (spn) (2), *S. sanguinis* (ssa) (3), *Lactococcus lactis* (lla) (4), *Enterococcus faecalis* (efa) (5), *Staphylcoccus aureus* (sau) (6), *Bacillus subtilis* (bsu) (7) and *Listeria monocytogenes* (lmo) (8) was produced by ClustalO. A key denoting residues as identical (*), conservative (:), semi-conservative (.), and non-conservative ( ) is located above the residues for comparison of SpxA1- or SpxA2-homologues, or below the residues for comparison of all sequences. Boxed residues include the N-terminal CxxC redox sensing domain and the G52 residue critical for interaction with RNA Polymerase in *B. subtilis*.

**Figure S3. ClpX positively regulates *slo* expression and over-expression of SpxA2, but not SpxA1, suppresses expression of SpeB. (A)** Expression of the gene encoding Streptolysin O (L897_00955) was analyzed in the indicted mutants by real time RT-PCR, as previously described (9) at the mid- (50% of final OD_600_) or late-logarithmic phase (75% of final OD_600_) of growth in C-medium. Data presented represent the mean and standard error of the mean derived from at least 3 independent experiments. **(B)** Plasmid vector alone (pVector), or vector expressing SpxA1 (pSpxA1) or SpxA2 (pSpxA2) (see Supplemental methods) were introduced into the WT strain (HSC5). Overnight cultures were serially diluted and aliquots plated on protease indicator media (dilution) which were imaged following an additional 24hr of incubation, as shown. SpeB protease activity is apparent as a zone of clearing around the area of bacterial growth. As described (5), the relative level of SpeB expression corresponds to the highest dilution for which a zone of clearing is observed. Strains: pVector, GCP017; pSpxA1, ZC572; pSpxA2, ZC573 (Table S1).

**Figure S4. Growth under aerobic conditions is bacteriostatic.** Indicated strains were cultured in liquid media for 48 hours under anaerobic (-O_2_) or aerobic (+O_2_) conditions and growth was measured by determination of CFUs. After the first 24 hours of growth, conditions were either shifted or left the same as indicated at the right of Fig. by the arrow; anaerobic to anaerobic **(A),** anaerobic to aerobic **(B),** aerobic to aerobic **(C),** or aerobic to anaerobic **(D).**

**Figure S5. SpxA2**^-^ **hypervirulence is evident early in infection.** Hairless SKH1 mice were infected subcutaneously with 10^7^ CFU of indicated strains. Areas of the resulting ulcers were analyzed 24 hrs post-infection. Data are pooled from at least 2 independent experiments, with the mean indicated by a bar. Differences between wild type and mutant strains were tested for significance using the Mann-Whitney test (***, P < 0.001; ns, not significant).

**Table S1. Bacterial strains utilized in study**

**Table S2. Plasmids utilized in study**

**Table S3. Primers utilized in study**

**Table S4. Growth rates and yields for various *S. pyogenes* mutants**.

**SUPPLEMENTARY FIGURE REFERENCES**

1. Kajfasz JK, Martinez AR, Rivera-Ramos I, Abranches J, Koo H, Quivey RG, Jr., Lemos JA. 2009. Role of Clp proteins in expression of virulence properties of *Streptococcus mutans*. J Bacteriol 191:2060-8.

2. Turlan C, Prudhomme M, Fichant G, Martin B, Gutierrez C. 2009. SpxA1, a novel transcriptional regulator involved in X-state (competence) development in *Streptococcus pneumoniae*. Mol Microbiol 73:492-506.

3. Chen L, Ge X, Wang X, Patel JR, Xu P. 2012. SpxA1 involved in hydrogen peroxide production, stress tolerance and endocarditis virulence in *Streptococcus sanguinis*. PLoS One 7:e40034.

4. Duwat P, Ehrlich SD, Gruss A. 1999. Effects of metabolic flux on stress response pathways in *Lactococcus lactis*. Mol Microbiol 31:845-58.

5. Kajfasz JK, Mendoza JE, Gaca AO, Miller JH, Koselny KA, Giambiagi-Demarval M, Wellington M, Abranches J, Lemos JA. 2012. The Spx regulator modulates stress responses and virulence in *Enterococcus faecalis*. Infect Immun 80:2265-75.

6. Pamp SJ, Frees D, Engelmann S, Hecker M, Ingmer H. 2006. Spx is a global effector impacting stress tolerance and biofilm formation in *Staphylococcus aureus*. J Bacteriol 188:4861-70.

7. Nakano MM, Hajarizadeh F, Zhu Y, Zuber P. 2001. Loss-of-function mutations in yjbD result in ClpX- and ClpP-independent competence development of *Bacillus subtilis*. Mol Microbiol 42:383-94.

8. Borezee E, Msadek T, Durant L, Berche P. 2000. Identification in *Listeria monocytogenes* of MecA, a homologue of the *Bacillus subtilis* competence regulatory protein. J Bacteriol 182:5931-4.

9. Port GC, Vega LA, Nylander AB, Caparon MG. 2014. *Streptococcus pyogenes* polymyxin B-resistant mutants display enhanced ExPortal integrity. J Bacteriol 196:2563-77.
