# Supplementary material for: SpxA1 and SpxA2 Act Coordinately To Fine-Tune Stress Responses and Virulence in Streptococcus pyogenes
Source: mBio. 2017 Mar 28;8(2):e00288-17. doi: 10.1128/mBio.00288-17 (PMC5371413; doi:10.1128/mBio.00288-17)
Supplement: TABLE S2 [file mbo002173246st2.pdf]

Table S2. Plasmids used in this study

Plasmid [alternate name] (resistance)[a]	Features	Reference
**Cloning plasmids**		
pABG5 [pVector] (Kan, Cam)	Shuttle vector	(1)
pJRS233 (Erm)	Low-copy temperature-sensitive shuttle vector used for allelic replacement	(2)
pGCP213 (Erm)	High-copy temperature-sensitive shuttle vector used for allelic replacement	(3)
pSPC18 (Spc)	Integrational vector	(4)
**Allelic replacement plasmids**		
pGCP666 (Erm)	pGCP213::Δ*clpX*, allelic replacement plasmid	(5)
pGCP1289 [pZC165] (Erm)	pJRS233::Δ*spxA1*, allelic replacement plasmid	This study
pGCP1001 (Erm)	pJRS233::*clpX*, allelic replacement complementation plasmid	This study
pGCP1290 [pZC192] (Erm)	pJRS233::Δ*spxA2*, allelic replacement plasmid	This study
pGCP485 [pCK365] (Erm)	pGCP213::Δ*speB*, allelic replacement plasmid	This study
**Insertional disruption plasmids**		
pGCP647 (Spc)	pSPC18::Ω*clpP*, insertional disruption plasmid	This study
pGCP648 (Spc)	pSPC18::Ω*clpL*, insertional disruption plasmid	This study
pGCP649 (Spc)	pSPC18::Ω*clpE*, insertional disruption plasmid	This study
pGCP651 (Spc)	pSPC18::Ω*ctsR*, insertional disruption plasmid	This study
pGCP650 (Spc)	pSPC18::Ω*clpC*, insertional disruption plasmid	This study
pWAR251 [pGCP1291] (Erm, Spc)	pGCP213::Δ*clpP*::*aad9*, allelic replacement complementation plasmid	(6)
**Complementation plasmids**		
pGCP893 [pClpX, pZC154] (Kan, Cam)	pABG5::ClpX, expression vector	(5)
pGCP610 (Erm)	pJRS233::3'*guaB*-*clpX*, allelic replacement plasmid	This study
pJL60 (pGCP694)	pABG5::RopB-HA, expression vector	(7)
pZC169 (pSpxA1-6xHis) (Kan, Cam)	pABG5::SpxA1-6xHis, expression vector	This study
pZC170 (pSpxA2-6xHis) (Kan, Cam)	pABG5::SpxA2-6xHis, expression vector	This study
**Ectopic chromosomal complementation plasmid intermediates**		
pET28sfGFP [pGCP059] (Kan)	source of *sfGFP*	(8)
pGCP140b (Erm)	pJRS233::3'GuaB region, used to generate pGCP189	This study
pGCP189 (Erm)	pJRS233::3'GuaB-*sfGFP*, used to generate pGCP610	This study

[a]Antibiotics are abbreviated as follows: kanamycin (Kan), chloramphenicol (Cam), spectinomycin (Spc), erythromycin (Erm).

REFERENCES

1. Granok AB, Parsonage D, Ross RP, Caparon MG. 2000. J Bacteriol. Mar;182(6):1529-40.
2. Perez-Casal J, Price JA, Maguin E, Scott JR. 1993. Mol Microbiol. May;8(5):809-19.
3. Nielsen HV, Guiton PS, Kline KA, Port GC, Pinkner JS, Neiers F, Normark S, Henriques-Normark B, Caparon MG, Hultgren SJ. 2012. Mbio. Jul 24;3(4):e00177-12.
4. Cho KH, Caparon MG. 2005. Mol Microbiol. Sep;57(6):1545-56.
5. Port GC, Vega LE, Nylander AB, Caparon MG (2014) *Streptococcus pyogenes* polymyxin B-resistant mutants display enhanced ExPortal integrity. J Bacteriol Jul;196(14):2563-77.
6. Mashburn-Warren L, Morrison DA, Federle MJ. 2012. J Bacteriol. Sep;194(17):4589-600.
7. Loughman JA, Caparon M (2007) Contribution of invariant residues to the function of Rgg family transcription regulators. J Bacteriol 189: 650-655.
8. Pédelacq J-D, Cabantous S, Tran T, Terwilliger TC, Waldo GS. 2006. Nat. Biotechnol. 24:79-88.
