# Supplementary material for: SpxA1 and SpxA2 Act Coordinately To Fine-Tune Stress Responses and Virulence in Streptococcus pyogenes
Source: mBio. 2017 Mar 28;8(2):e00288-17. doi: 10.1128/mBio.00288-17 (PMC5371413; doi:10.1128/mBio.00288-17)
Supplement: TABLE S3 [file mbo002173246st3.pdf]

Table S3 Mutagenesis, complementation and RT-PCR primers used in this study

Name (description)[a]	Sequence[b]	Template	Plasmid[c]
GP728 (ΩClpP-M13F-F1)	tgtaaaacgacggccagt-GACCGTATTATCATGTTAACAGG	HSC5	pGCP647
GP729 (ΩClpP-M13R-R1)	cacacaggaaacagctatgac-ATTGGCTGGTGGATCATATAC		
GP730 (ΩClpL-M13F-F1)	tgtaaaacgacggccagt-CGAAGAATTCCAACACTATCG	HSC5	pGCP648
GP731 (ΩClpL-M13R-R1)	cacacaggaaacagctatgac-GGACATCTCCATTAACAATGG		
GP732 (ΩClpE-M13F-F1)	tgtaaaacgacggccagt-GAGCACAAGATAGATCCACAAG	HSC5	pGCP649
GP733 (ΩClpE-M13R-R1)	cacacaggaaacagctatgac-CTCGATAACGCGTGTAATCTC		
GP734 (ΩClpC-M13F-F1)	tgtaaaacgacggccagt-CCATAGAAGAATATGAAGCTGC	HSC5	pGCP650
GP735 (ΩClpC-M13R-R1)	cacacaggaaacagctatgac-CTGCATGACGTTCTATTGC		
GP736 (ΩCtsR-M13F-F1)	tgtaaaacgacggccagt-GCAGAAATTAAGCGGTCC	HSC5	pGCP651
GP737 (ΩCtsR-M13R-R1)	cacacaggaaacagctatgac-CCTCACGCTCTGTCAATAAG		
GP712 (ClpX-M13F-F1)	tgtaaaacgacggccagt-GAGGTGTTGACAATGACTAGTATTG	HSC5	pGCP1001
GP715 (ClpX-M13R-R1)	cacacaggaaacagctatgac-ACCTATCGCTAGACCTGCTC		
GP258 (5'GuaB-BamHI-F1)	GCT-GGATCC-CTATGAAGAAAGGATCAAGCG	HSC5	pGCP140b
GP206 (5'GuaB-ClaI-P-R2)	gCT-ATCGAT-AATTAAATTATCAGTGCACAGAATAG		
GP207 (3'GuaB-PstI-P-F3)	gCT-CTGCAG-TAGTAACTTTAAGGGCTGACATATTG		
GP259 (3'GuaB-KpnI-R4)	GCT-GGTACC-AGTCATTCCTAGAGTTTGTTGC		
GP209 (sfGFP-ClaI-F)	GCT-ATCGAT-*AAGAAGGAGATATACATATGAGCAAAG*	pGCP059	pGCP189
GP038 (sfGFP-PstI-R)	CCC-CTGCAG-*TTATTTGTAGAGCTCATCCATGC*		
GP687 (3'GuaB-ClpX^{R}-F)	*gcactgataatttaattatcgataagaaggagatatacat*-ATGGCAGGAAATAGAACTAACG	HSC5	pGCP610
GP688 (3'GuaB-ClpX^{R}-R)	*caatatgtcagcccttaaagttactactgcagtta*-AGCTGTCTCTAAAACGGG		
CK550 (ΔSpeB-BamHI-F1)	CCC-GGATCC-GATAAATCAGCCAGGAAAAAGCC		pGCP485 (pCK365)
CK549 (ΔSpeB-R2)	tGCACCTGCTTTGATAGCTGCTG		
CK548 (ΔSpeB-F3)	gATGGTGCTGACGGACGTAAC		
CK547 (ΔSpeB-KpnI-R4)	CCC-GGTACC-GCGTTGATGACCTCCTTTATC		
ZC374 (ΔSpxA1-SalI-F1)	CCCCTCGAG-GTCGAC-GAAGATCATCGAAAAATTTCTGCTACATG	HSC5	pGCP1289 (pZC165)
ZC375 (ΔSpxA1-R2)	TTTC-GCTAGGTGATAAAAATAAGGTAACCATATTTTT		
ZC376 (ΔSpxA1-F3)	GAAATTGAGGGGTGATATGTCAGG		
ZC377 (ΔSpxA1-PstI-R4)	CCCCCGGG-CTGCAG-GATTAATGGGGTGACGAACGTC		
GP799 (ΔSpxA2-M13R-F1)	cacacaggaaacagctatgac-GACTACTGTTCATAGGTGTGTGG	HSC5	pGCP1290 (pZC192)
ZC380 (ΔSpxA2-R2)	GCTCGTACAACTTGAAATCGTG		
ZC379 (ΔSpxA2-F3)	CACGATTTCAAGTTGTACGAGC-GCACGATTACGTGCTGCAC		
GP800 (ΔSpxA2-M13F-R4)	tgtaaaacgacggccagt-CCCACTCAAGACATAACCAAC		
ZC388 (pSpxA1-6xHis-EcoRI-F)	AACT-GAATTC-ATGGTTACCTTATTTTTATCACCTAGCTGTACC		
ZC392 (pSpxA1-6xHis-PstI-R)	TGAT-CTGCAG-*TCAGTGGTGGTGGTGGTGGTG*-CCCCTCAATTTCTGCTTTTATCGTTGCTTGACG	HSC5	pZC169
ZC390 (pSpxA2-6xHis-EcoRI-F)	AACT-GAATTC-ATGATTAAAATTTACACGATTTCAAGTTG		pZC170
ZC393 (pSpxA2-6xHis-PstI-R)	TGAT-CTGCAG-*TTAGTGGTGGTGGTGGTGGTG*-GAGTGCAGCACGTAATCGTGCTTCAG		
GP1021 (*recA*-F)	AGTGATGCGATTAGGAGAACG		
GP1022 (*recA*-R)	TCGTTTTACCGGAAGACTCTG		
GP1023 (*speB*-F)	CCAAGGTGTCGGTAAAGTAGG		
GP1024 (*speB*-R)	AGAGCTGAAGGGTTTAGTGC		
JLP29 (*ropB*-F)	TGAACGGTGTTGTGTGTCTTT		
JLP30 (*ropB*-R)	TGGATCGTTTTGCAATTGAG		
CK361 (*slo*-F)	GCTAGTACAGAAACCACAAC		
CK362 (*slo*-R)	CTAGTGGCATTTCTTTGGGAG		

[a]Primers are categorized as forward (F) or reverse (R) relative to the direction of transcript.

[b]Sequence is shown 5' to 3'. Uppercase sequence anneals to the HSC5 chromosome, uppercase italics sequence anneals to the *sfGFP* gene, uppercase underlined italics sequence introduces a 6x-His tag, lowercase sequence anneals to the M13F and M13R universal primer sequences, lowercase italics sequence anneals to the parental plasmid. Hyphens indicate junctions between contiguous DNA regions, uppercase underline indicates restriction sites, lowercase underline indicates phosphorylated 5' ends.

[c]Plasmid that was constructed using the indicated primers.
