# Supplementary material for: SpxA1 and SpxA2 Act Coordinately To Fine-Tune Stress Responses and Virulence in Streptococcus pyogenes
Source: mBio. 2017 Mar 28;8(2):e00288-17. doi: 10.1128/mBio.00288-17 (PMC5371413; doi:10.1128/mBio.00288-17)
Supplement: TABLE S4 [file mbo002173246st4.pdf]

Table S4. Growth rates and yields for various *S. pyogenes* mutants.

Strains (alternate name)	Relevant Genotype	Growth Rate[a] (% yield)[b] ThyB	C Medium
HSC5	wild type (WT)	43.8 (100)	50.5 (100)
GCP688	ClpX⁻	52.9 (93)	67.9 (104)
GCP1245 (ZC611)	SpxA1⁻	42.0 (83)	54.2 (102)
GCP1033	SpxA2⁻	41.4 (81)	50.2 (101)
GCP711	ClpX⁻/SpxA1⁻	58.0 (86)	72.0 (105)
GCP726	ClpX⁻/SpxA2⁻	41.1 (83)	50.1 (105)
GCP729	ClpX⁻/SpxA1⁻/SpxA2⁻	85.1 (51)	97.3 (85)
GCP1072	SpxA1⁻/SpxA2⁻	74.5 (62)	83.0 (99)
GCP652	ΩClpP	60.8 (101)	94.7 (75)
GCP1300	ClpP::*aad9*	72.9 (103)	91.0 (88)

[a]Growth rates (doubling time in min.) are calculated from early logarithmic growth in indicated medium.

[b]Yields (% of WT) are calculated from overnight growth in indicated medium in comparison to WT.
